# Mechanistic and Kinetic Investigations on the Ozonolysis of Biomass Burning Products: Guaiacol, Syringol and Creosol

**DOI:** 10.3390/ijms20184492

**Published:** 2019-09-11

**Authors:** Xiaoxiao Chen, Yanhui Sun, Youxiao Qi, Lin Liu, Fei Xu, Yan Zhao

**Affiliations:** 1 College of Environment and Safety Engineering, Qingdao University of Science and Technology, Qingdao 266042, China; 15621081790@163.com (X.C.); 15965425707@163.com (Y.Q.); liulin0505@163.com (L.L.); 2 State Key Laboratory of Pulp and Paper Engineering, South China University of Technology, Guangzhou 510640, China; 3 Environment Research Institute, Shandong University, Qingdao 266237, China; xufei@sdu.edu.cn; 4 School of Life Sciences, Qufu Normal University, Qufu 273165, China; zhaoyan08319@126.com

**Keywords:** biomass burning, guaiacol, syringol, creosol, reaction pathways, rate constant

## Abstract

The lignin pyrolysis products generated by biomass combustion make an essential contribution to the formation of secondary organic aerosols (SOAs). The ozone-initiated oxidation of guaiacol, syringol and creosol, major constituents of biomass burning, were investigated theoretically by using the density functional theory (DFT) method at the MPWB1K/6-311+G(3df,2p)//MPWB1K/6-31+G(d,p) level. Six primary addition reaction pathways and further decomposition routes with corresponding thermodynamic values were proposed. The Criegee intermediates can be excited by small molecules, such as NO_x_, H_2_O in the atmosphere, and would further proceed via self-decomposition or isomerization. The most predominant product for ozonation of guaiacol is the monomethyl muconate (P1). At 295 K and atmospheric pressure, the rate constant is 1.10 × 10^−19^ cm^3^ molecule^−1^ s^−1^, which is lies a factor of 4 smaller than the previous experimental study. The branching ratios of the six channels are calculated based on corresponding rate coefficient. The present work mainly provides a more comprehensive and detailed theoretical research on the ozonation of methoxyphenol, which aspires to offer novel insights and reference for future experimental and theoretical work and control techniques of SOAs caused by lignin pyrolysis products.

## 1. Introduction

Biomass refers to various organisms engendered by plants through photosynthesis. It is a renewable energy that is abundant, cheap and easy to obtain. Biomass combustion can be understood as open-air or quasi-open burning of any non-fossil plant or organic fuel in a broad sense [1]. It not only contributes nearly 90% of primary organic aerosols (POAs) to the earth [2], but also frees a deal of organic gases and steams, which promotes the formation of secondary organic aerosols (SOAs) [3]. Overall, biomass combustion is the important origin of primary fine carbonaceous particles and trace gases. Thus, it has posed an unpredictable and far-reaching threat to the global atmosphere [4] and climate change as well as human health [5]. Lignin is an ample constituent in biomass [6,7] that can be switched to commercially viable products through pyrolysis, such as biofuels and phenolic chemicals [8,9,10,11]. In recent years, lignin pyrolysis has stolen the spotlight from public. It has brought a lot of benefits, but has created some severe problem as well. Lignin burning produces extensive methoxyphenol [12], the most three of which are guaiacol, creosol and syringol in fresh wood smoke [13]. They mainly exist in the gas-phase since they have relatively high vapor pressure at 25 °C [14]. Once methoxyphenol enters into the atmosphere, it is prone to react with oxidants, particular with OH radicals, NO_3_ radicals, Cl atom and ozone molecules.

Given this, some studies have been performed clearly. The reaction of syringol with OH radicals was investigated by Lauraguais et al. [15]. They indicated that syringol is too active and degrades rapidly in the ambient air, which is associated with aerosol yield. Liu et al. [16] demonstrated that the yield of SOA is dependent on the concentration of guaiacol and OH radicals. Besides, the presence of SO_2_ and NO_2_ is beneficial to increase the SOA yield. Photo-oxidation experiments were carried out by Yee et al. [17] at low-NOx conditions. The yields of SOAs produced by the oxidation of phenol, guaiacol and syringol are greater than 25%. Yang et al. [18] illustrated the major degradation products of guaiacol, creosol and syringol with NO_3_ respectively and measured the corresponding reaction constants by using the relative rate method. These achievements provide guidance for approaching research work. 

Furthermore, ozone abounds with sources, and its concentration is far greater OH radicals [19]. Most notably, ozone is a toxic air pollutant with powerful oxidizing and strong irritant properties [20]. Therefore, the effect of ozone on methoxyphenol can not to be neglected in the atmosphere. Khudoshin et al. [21] analyzed the main oxidation products of guaiacol with ozone by high performance liquid chromatography (HPLC), and presented the reaction mechanisms of ozone with guaiacol. Unfortunately, although the mechanism they proposed is reasonable, it is not comprehensive enough and still needs to be supplemented. The reaction constant of guaiacol with ozone have been investigated by Zein et al. [22]. They determined that the value is (0.40 ± 0.31) × 10^−18^ cm^3^ molecule^−1^ s^−1^ at 294 ± 2 K, atmospheric pressure and dry conditions.

Nowadays, to clarify the impact of ozone on methoxyphenol in the atmosphere has become a task we must do. In recent researches, the gas-phase reaction mechanisms of guaiacol, syringol and creosol with ozone are insufficient. In order to gain a deeper understanding of the migration and transformation of methoxyphenol produced by biomass combustion, we presented initial reactions of guaiacol, syringol and creosol with ozone. Additionally, this work explored more profound reaction mechanisms and calculated the rate coefficient of guaiacol with ozone.

## 2. Results and Discussion

**Figure 1:**
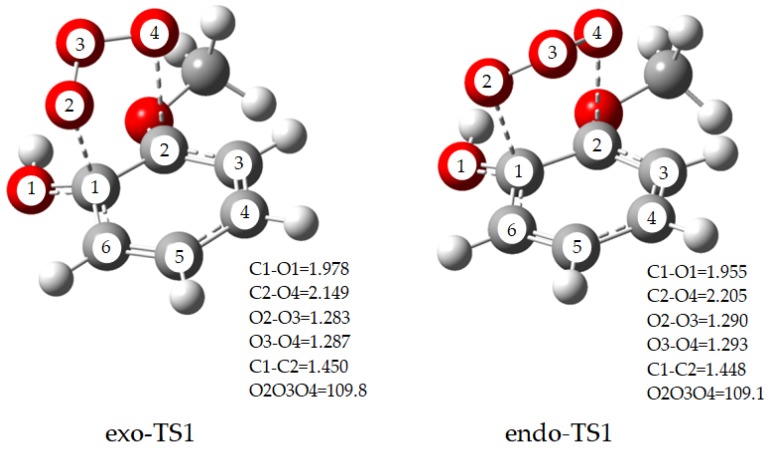
MPWB1K/6-31G(d,p) optimized geometries for exo-TS1and endo-TS1 of guaiacol with main bond lengths and bond angles. Angles are in degree and bond lengths are in Å.

As mentioned above, ozone is strong oxidant and methoxyphenol is not stable because of the unsaturated C=C double bond. Therefore, ozone reacts easily with methoxyphenol. Ozone addition at C=C double bond in benzene ring can form primary ozonides (POZs). Owing to the central O atom of ozone can point to or away from the aromatic ring, POZ has two different structures, and the corresponding transition state TS also has two conformers, namely endo-TS and exo-TS. For instance, Figure 1 depicts the MPWB1K/6-31+G(d,p) optimized geometries of the transition state TS1 by 1,3-cycloaddition of guaiacol with main bond lengths and bond angles. It is worth noting that endo-TS1 has a 4.97 kcal/mol lower barrier than exo-TS1. In Table S1 of Supplementary Materials, the energy barriers between the exo-TS and endo-TS of the six ozone-addition pathways of guaiacol, syringol and creosol were compared. The comparison reveals that in all the cases, the energy barrier of the latter (endo-TS) is lower than that of the former (exo-TS), and they have a gap of 1.61‒5.19 kcal/mol. Moreover, endo-TS is more favorable than exo-TS, which is consistent with the reported results of Barnum [23]. Consequently, the latter (endo-TS) is selected for the following study.

### 2.1. Initial Reaction of Guaiacol with Ozone

**Figure 2:**
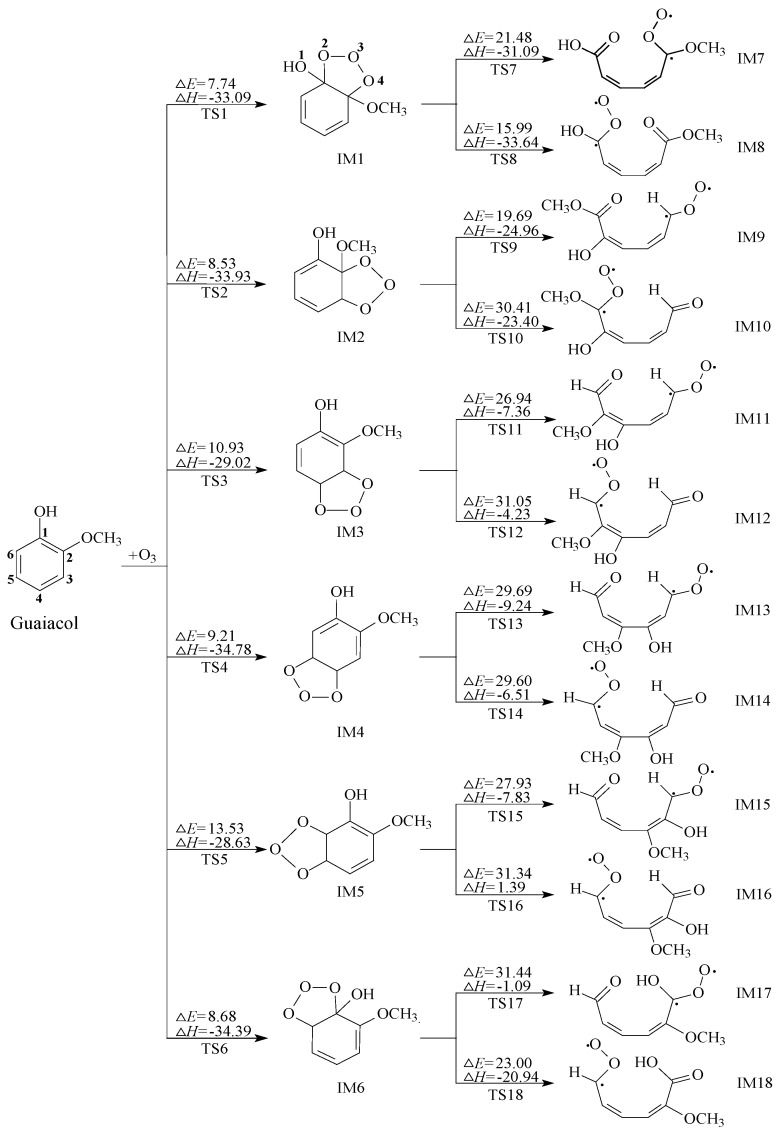
The initial reaction routes of ozone with guaiacol, ∆*E*: potential-energy barriers, ∆*H*: heats of reaction, TS: transition state, IM: intermediate.

**Figure 3:**
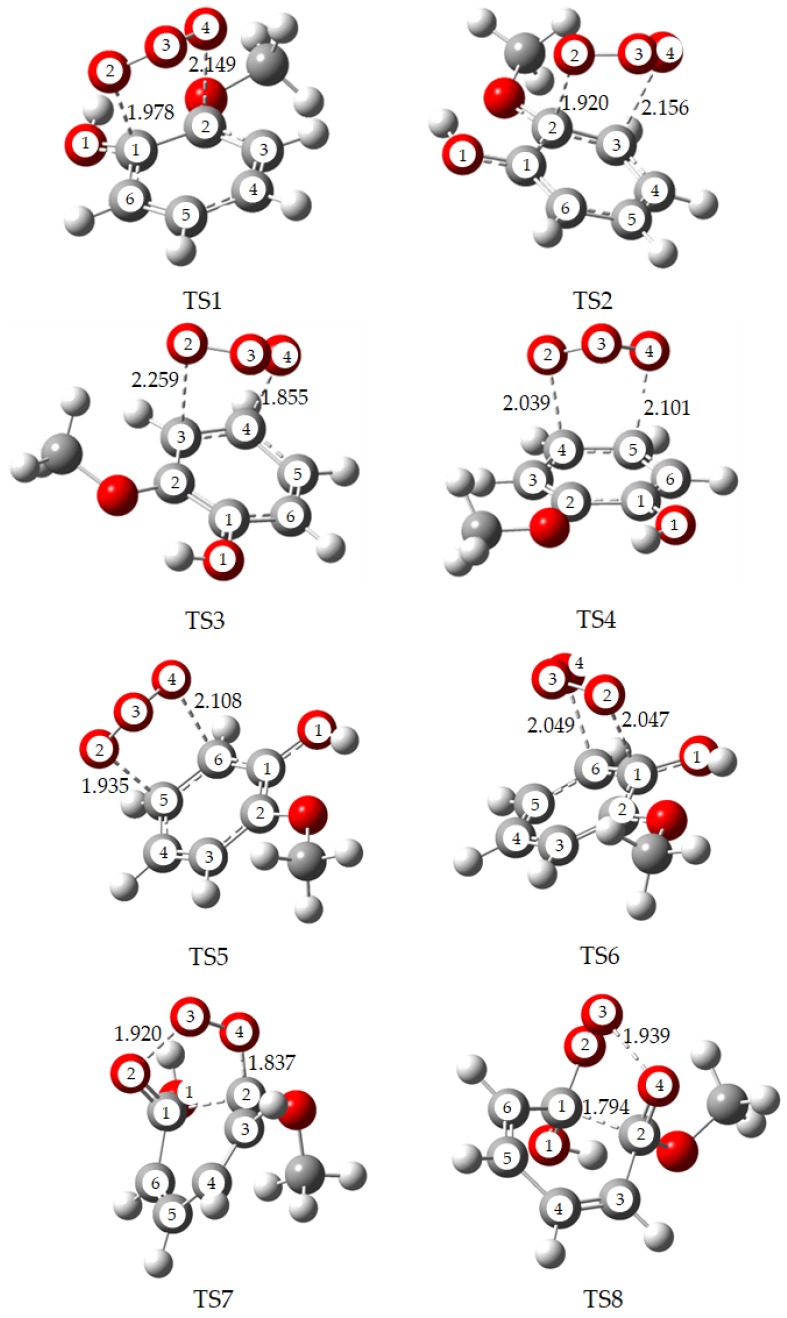
MPWB1K/6-31+g(d,p) optimized geometries for the transition states involved in the initial reaction of guaiacol + ozone with main bond lengths. Bond lengths are in Å.

For convenience, the labeling of the C atom and O atom in the benzene ring are illustrated in Figure 2. The orientation of OCH_3_ and OH groups bring about the structural asymmetry of guaiacol, thus there are six unequal addition sites on the benzene ring. Their specific reaction parameters are also depicted in Figure 2. The ozonolysis of guaiacol occurs in two outer O atoms of ozone simultaneously adding to two neighboring C atoms forming a five-membered ring. At first, ozone addition at site C1 produces primary ozonide IM1 via transition state TS1. The bond lengths of C1–O2, C2–O4 in IM1 are 1.433 and 1.423 Å, respectively. The corresponding bond in TS1 is elongated by 27.52% and 33.79% compared to IM1. Similarly, TS2, TS3, TS4, TS5 and TS6 are the transition states. IM2, IM3, IM4, IM5 and IM6 are the corresponding POZs. The structures of all transition states are presented in the Figure 3. The optimized geometries of all the primary ozonides, transition states and Criegee intermediates involved in reaction of guaiacol + ozone with main bond lengths are shown in Figure S1 of Supplementary Materials. The thermodynamic calculation displays that the activation energies of these routes are in the range of 7.74–13.53 kcal/mol, and the exothermic energy ranges from 28.63–34.78 kcal/mol. In all the addition channels of guaiacol, the route via transition state TS1 is the most favorable by reason of the lowest activation barrier. According to the previous reports [17,24,25], this is because OCH_3_, OH and CH_3_ groups belong to the electron-donating substituents, which enhance the density of the electron cloud at site C1. Hence, the first channel is thermodynamically most advantageous.

The POZs (IM1-IM6) are unstable, which will undergo the decomposition pathways. The cleavages of O2–O3 and O3–O4 bonds in the POZ result in the formation of two different Criegee intermediates. The two transition states of TS7 and TS8 (Their structures are also exhibited in the Figure 3) are generated by the ring-cleavage of IM1 confirmed by the intrinsic reaction coordinate (IRC) method, and IM7 and IM8 are also corresponding Criegee intermediates. It is seen from Figure 2 that the channel via transition state TS8 merely needs to overcome the lowest barrier of 15.99 kcal/mol and releases the highest energy of 33.64 kcal/mol. This process is the most favorable in decomposition pathways of all POZs. Specifically, the lengths of the C1–C2 and O3–O4 bonds in TS8 are 1.794 and 1.939 Ǻ, respectively. What’s more, Criegee intermediate IM8 is the easiest to generate.

**Figure 4:**
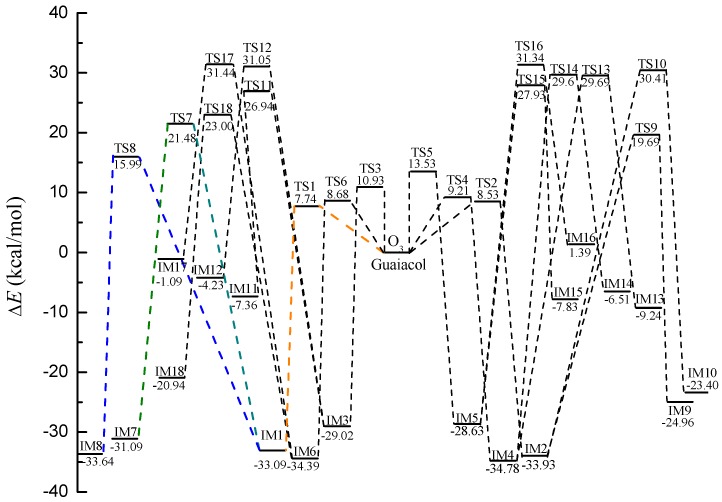
Profiles of the energy surface for the initial reaction of guaiacol with ozone, and the followed self-decomposition.

Criegee intermediates are labile and can react with H_2_O, NO_x_ in the atmosphere to take place subsequent reactions, or undergo self-decomposition or isomerization. In the following section, IM8 is chosen as representative to explore their secondary reactions. The reaction mechanisms of the other Criegee intermediates (IM7, IM9-IM18) are similar to IM8. Figure 4 exhibits the potential energy surfaces of all the POZs and Criegee intermediates, emerging the degree of difficulty at each reaction pathway in a more intuitive manner.

### 2.2. Secondary Reactions of IM8

**Figure 5:**
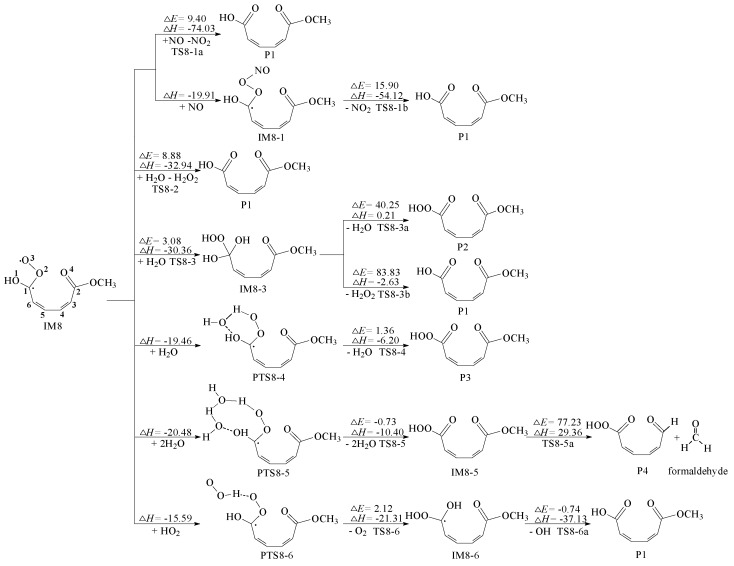
Secondary reactions of IM8 with some small molecules, ∆*E*: potential-energy barriers, ∆*H*: heats of reaction, TS: transition state, IM: intermediate, P: product.

As stated above, we will debate secondary reactions of IM8 in this section, which is the top priority of this article. IM8 has the capability to react with some small molecules, especially NO, H_2_O molecule and HOO radical as illustrated in Figure 5.

To begin with, we studied the reaction pathways of IM8 with NO_x_. There are two channels, which produce energetically most advantageous product monomethyl muconate (P1) in the secondary reactions of IM8. The O3 atom of IM8 is extracted by the NO molecule to form P1 and NO_2_ through transition state TS8-1a, which is the first channel. The potential barrier is 9.40 kcal/mol and the reaction heat is −74.03 kcal/mol. The second process has two steps. At first, the N atom of the NO molecule is attached to the O3 atom of IM8 to generate IM8-1, which is a barrierless route and exothermic energy of 19.91 kcal/mol; Then from IM8-1 to P1, the cleavage of O2–O3 bond is accompanied by the elimination of NO_2_, which has an energy barrier of 15.90 kcal/mol and releases 54.12 kcal/mol of energy. Both the two channels are easy to occur.

Besides, for the reactions of IM8 with H_2_O, four possible pathways are included in Figure 5. The first pathway is to add H_2_O with the elimination of H_2_O_2_ via transition state TS8-2, which generates the most dominant product P1. In TS8-2, the O2–O3 bond length is 3.294 Å, whereas the distance of O3 atom with the O atom of H_2_O is 1.560 Å. The angle between O2, O3 and the O atom of H_2_O is 52.30 degree. This process is exothermic by 32.94 kcal/mol with an estimated barrier of 8.88 kcal/mol, which is the lowest accessible channel. The second pathway is to transfer one H atom in H_2_O to the O3 atom, while the remaining OH group of H_2_O molecule is attached to C1 forming IM8-3. This process is exothermic by 30.36 kcal/mol with an energy barrier of 3.08 kcal/mol. Moreover, IM8-3 is unstable which tends to proceed following two routes. The first route is that two OH group in C1 take off H_2_O producing P2. Another route is that the OH group in the OOH group removes together with one of OH group at C1. Both the two routes can hardly take place owing to the high barrier. The following-up two pathways generate van der Waals complexes PTS8-4 and PTS8-5 respectively, corresponding to the release of 19.46 and 20.48 kcal/mol of heat. Herein the H_2_O molecule acts as a “water bridge”, which transfers the H atom on the OH group in C1 to the peroxy radical leading to OOH group. The energy barrier and endothermic energy of the first path are 1.36 and 6.20 kcal/mol, respectively. Similarly, the energy barrier of the other path is −0.73 kcal/mol, and endothermic energy is 10.40 kcal/mol. Both the two paths are prone to occur. The above analysis indicates that the energy barrier of the initial reaction of IM8 and H_2_O is generally lower, but that of the further reaction is higher.

Additionally, peroxy radical intermediates are able to react with other peroxide groups (HO_2_ and RO_2_, where R is a carbon-containing group) [26,27]. The intermediate PTS8-6 is formed by the reaction of IM8 with HOO radical. Eliminating O_2_ of PTS8-6 has an energy barrier of 2.12 kcal/mol and exothermic of 21.31 kcal/mol. It’s worth noting that the successive step is accompanied by the formation of P1. The potential barrier is −0.74 kcal/mol with reaction heat of −37.13 kcal/mol, which is likely to occur.

After the above analysis, the secondary reactions of IM8 with small molecules are relatively easy to carry out in general. IM8 not only have these reaction pathways, but also undergoes its own rearrangement, isomerization and decomposition.

**Figure 6:**
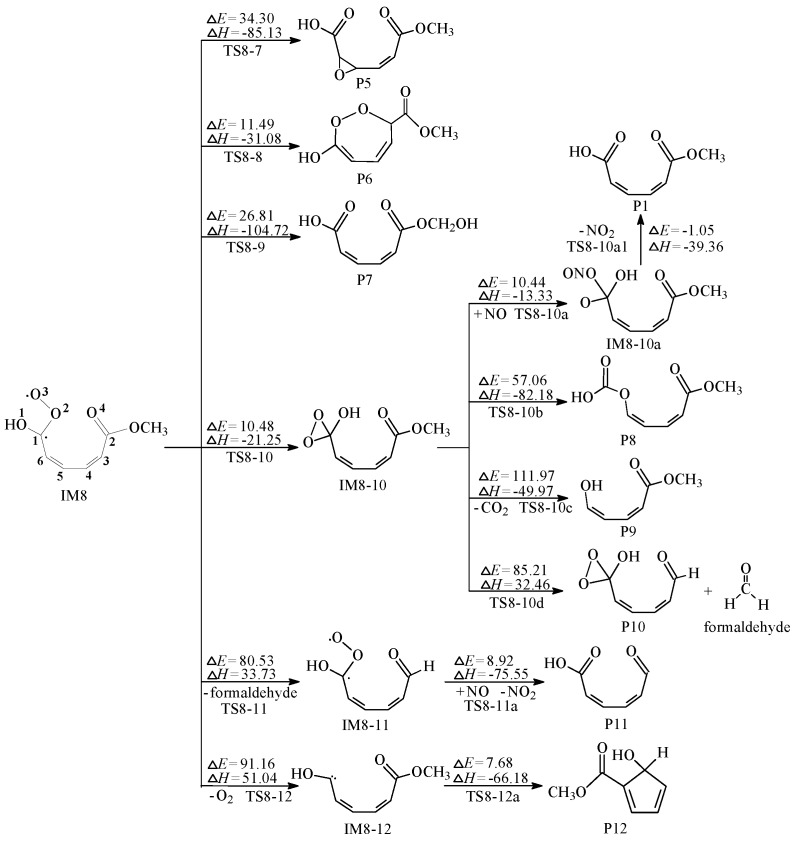
The rearrangement and self-decomposition processes of IM8, ∆*E*: potential-energy barriers, ∆*H*: heats of reaction, TS: transition state, IM: intermediate, P: product.

Rearrangement reactions of IM8 are displayed in Figure 6. The first channel produces P5 by adding the O3 atom to both ends of C6=C5 double bond. This way is capable of proceeding because of moderate energy barrier (34.30 kcal/mol) and high heat release (85.13 kcal/mol). The second channel is to render the cyclization of IM8 by the formation of C3–O3 bond, namely via the transition state TS8-8 to produce P6. The bond lengths of O2–O3, C3–O3 in TS8-8 are 1.531 and 2.408 Å, while the corresponding lengths in P6 are 1.431 and 1.390 Å. What’s more, this way is likely to occur because it releases 31.08 kcal/mol with an expected barrier of 11.49 kcal/mol. The last channel describes that O3 atom attacks the C atom in OCH_3_ group and simultaneously one H atom on the OCH_3_ group shifts to O3 atom generating OH group, which has moderate barrier (26.81 kcal/mol) and strong exothermic property (104.72 kcal/mol). Both the three channels are able to carry on.

Furthermore, the isomerization processes of IM8 are shown in Figure 6. IM8 has an unpaired single electron in C1 and O3 atoms respectively, which generates IM8-10 by O3 atom connecting with C1 atom. This channel needs to go through the potential barrier of 10.48 kcal/mol and is exothermic by 21.25 kcal/mol, which is easy to carry out and move on to the follow-up pathways. The first pathway has two steps. The N atom of the NO molecule is connected to one of O atom of C1 to generate IM8-10a. Then IM8-10a eliminates NO_2_ to produce P1. Both the two steps could readily occur in the atmosphere. The second pathway is to produce P8 by destroying C1–C6 bond in IM8-10 and generating C–O bond. The third pathway is the self-decomposition of IM8-10, which produces CO_2_ and P9. Both the two pathways have the high reaction-energy barrier, which are virtually difficult to occur. The last pathway forms P10 and formaldehyde, which has difficulty in performing since high barrier (85.21 kcal/mol) and strong endothermicity (32.46 kcal/mol).

The ultimate two routes are the decomposition of IM8. Firstly, the reaction pathway from IM8 to IM8-11 is similar to that from IM8-10 to P10 and IM8-5 to P4. They are all involved in the formation of formaldehyde, which are almost impossible thanks to the existence of high energy barriers [28]. Following thermodynamic values of the three routes, we can assume that formaldehyde may be not easily produced in the reaction of ozone with guaiacol. Besides, from IM8-11 to IM8-11a, however, it has a significantly lower barrier of 8.92 kcal/mol and highly exothermic of 75.55 kcal/mol. It can demonstrate that NO molecule play an important role in the removal and transformation of organic pollutants in the atmosphere [20]. Another route is O_2_-elimination process, which has high barrier (91.16 kcal/mol). Still its isomerization route is easy to occur, which has a lower energy barrier (7.68 kcal/mol) and releases a large amount of heat (66.18 kcal/mol). We infer when a C atom has a diradical, this reaction is easy to occur. In consequence, the decompositions of IM8 are almost impossible to reach. MPWB1K/6-31+g(d,p) optimized geometries for the transition states, intermediates and products with respect to the secondary reactions of IM8 are provided in Figure S2.

### 2.3. Further Reaction of P1

**Figure 7:**
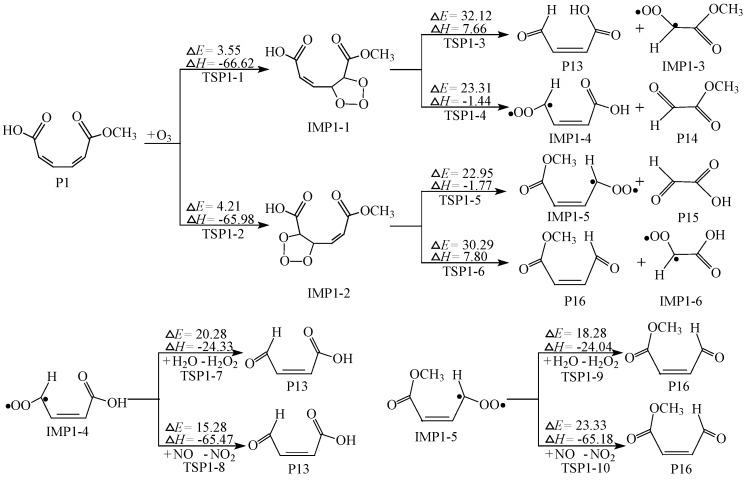
Further reaction of P1, ∆*E*: potential-energy barriers, ∆*H*: heats of reaction, TS: transition state, IM: intermediate, P: product.

As stated in the previous paragraph, P1 (monomethyl muconate) is the most advantageous product of all secondary reactions. But P1 has two unsaturated C=C double bonds and ozone still exists in the air, which may go a further reaction. There are two typical follow-up reaction pathways, namely the addition of ozone to two C=C double bonds respectively. The activation energies calculated are 3.55 kcal/mol for TSP1-1 and 4.21 kcal/mol for TSP1-2. The two processes are highly exothermic, with the reaction energies of 66.62 and 65.98 kcal/mol for IMP1-1 and IMP1-2, respectively. From Figure 7, it′s clear that the two processes through the transition states TSP1-4 and TSP1-5 are more advantageous than others among all the ring-cleavage pathways. Therefore, the two pathways were selected to explore further reaction routes. IMP1-4 and IMP1-5 are still unstable due to their biradical. In consideration of the thermodynamic values of the secondary reactions, we chose two channels that are most prone to occur, including the reaction of adding H_2_O to remove H_2_O_2_ group and extracting O atom with NO molecule. Hence, just take the two channels as examples to display the process of stabilization of IMP1-4 and IMP1-5 respectively.

In conclusion, the ozonolysis of monomethyl muconate (P1) generates four different products: Z-4-oxobut-2-enoic acid (P13), methyl 2-oxoacetate (P14), 2-oxoacetic acid (P15) and methyl (Z)-4-oxobut-2-enoate (P16). The products of ozonation of guaiacol are monomethyl muconate, muconic acid, and hydroquinone derivatives under certain experimental conditions [21]. It’s pity that we just calculated the reaction mechanism for the formation of monomethyl muconate (P1). Our theoretical research needs further expansion and verification. The geometries of all transition states, intermediates and products involved in further reaction of P1 are presented in Figure S3.

### 2.4. Kinetic Calculations

#### 2.4.1. Rate-constant Calculations

The transition state theory (TST) and canonical variational transition state theory (CVT) combined with zero-curvature tunneling (ZCT) or small-curvature tunneling (SCT) contributions, which have an increasingly wide utilization in calculating rate coefficients of many reactions [29,30,31,32,33]. This method (CVT/SCT) was utilized to calculate the reaction constants of the six preliminary pathways for ozone addition to guaiacol over a suitable temperature range (170–370 K).

The individual rate coefficient of six preliminary pathways are represented by *k*_i_ (i = 1–6) respectively. For example, *k*_1_ is the rate constant for ozone addition at site C1. In addition, *k*_tot_ refers to the total rate constant, where *k*_tot_ = ∑*k*_i_. The overall and individual rate constants at 1 atm and in the temperature range of 170–370 K are listed in Table 1. All of the individual reaction constants have the same trend that the calculated value increases as the temperature range from 170 to 370 K. That is to say the total rate coefficient *k*_tot_ also grows with increasing temperature. The range of *k*_tot_ is 4.60 × 10^−24^ to 2.23 × 10^−18^ cm^3^ molecule^−1^ s^−1^, when the temperature is raised from 170 to 370 K at 1 atmospheric pressure. The identified total rate constants of guaiacol with ozone is 1.10 × 10^−19^ cm^3^ molecule^−1^ s^−1^ at 295 K, which is in marked agreement with the previous experimental values of (0.40 ± 0.31) × 10^−18^ as investigated by Zein and co-workers [22].

Go in detail, as can be seen in Table 1, the rate coefficient for path 1, *k*_1_, is significantly bigger than others (path 2–path 6), and is rather near to the total rate constant *k*_tot_. The path 1 is fastest in accordance with our thermodynamic calculated results. On the contrary, the value of *k*_5_ is four to eight orders of magnitude lower than *k*_tot_. Thus, the path 5 can be neglected. In addition, for the pathway from IM1 to IM8, *k*_8_ is 7.50 × 10^−22^ cm^3^ molecule^−1^ s^−1^ at 295 K and atmospheric pressure.

#### 2.4.2. Branching Ratio

The rate constants as above provided, the branching ratio for the *i*th routes, *r*_i_, is calculated using the following equation:

(1) $$r_{i}=\frac{k_{i}}{k_{tot}}$$

The branching ratios within the determined temperature range are tabulated in Table 2. At 295 K, *r*_1_ is approached to 79%, which means that the initial addition routes of ozone with guaiacol is mainly carried out at site C1. The yield of IM2 is roughly 12%, placed second. The branching ratios for the formation of IM3, IM4, and IM6 are indicated to be 0.10%, 1.56%, 9.43% respectively, yet that of IM5 is insignificant as expected. Below 295 K, the ratio of R1 is found in high yields of more than 80%. In the spite of the branching ratio of R1 decreases with temperature, it still dominates the whole reaction.

#### 2.4.3. Atmospheric Implications

To assess the atmospheric implications of guaiacol emissions, the lifetime with respect to guaiacol with ozone reaction can be deduced from the total rate constant and the concentrations of ozone. The equation is determined as follows:

(2) $$\tau_{O_{3}}=\frac{1}{k_{O_{3}}\left[O_{3}\right]}$$

where *k* is the total rate coefficient for the reaction of guaiacol with ozone and [O_3_] is the average ozone concentration.

Taking into account the typical atmospheric ozone concentration (7 × 10^11^ molecules cm^−3^) [34], the calculated lifetime of guaiacol is 150.31 days at 295 K and 1 atm. Additionally, assuming an average ozone concentration of 100 ppbv (2.46 × 10^12^ molecules cm^−3^) at polluted atmosphere [35], the corresponding atmospheric lifetime of guaiacol is 42.77 days. However, the OH-determined lifetime of guaiacol is calculated to be 49.96h when the global average concentration of OH radicals is 10^6^ molecules cm^−3^ at 298 K and atmospheric pressure [36]. The lifetime of guaiacol in the typical atmospheric ozone concentration and the ozone concentration in the polluted area is higher than OH-determined lifetime. Therefore, the reactivity of the OH radicals is stronger than ozone and the removal of guaiacol by ozone cannot be neglected.

### 2.5. Initial Reactions of Syringol and Creosol with Ozone

The reaction mechanisms of guaiacol with ozone have been analyzed and described in detail. Additionally, there are two typical methoxyphenols, syringol and creosol, which have not been studied. Their structures are given in Figure S4. The structure of syringol is highly asymmetric since OH and one of OCH_3_ groups form intramolecular hydrogen bonding. There are six diverse addition routes on the aromatic ring. Their detailed reaction parameters are given in Figure S5. The activation energy of ozone addition to syringol is inferred to be in the range of 5.32–10.08 kcal/mol, and the exothermic energy is 31.51–37.38 kcal/mol. IM6 is the most stable POZ due to the lowest energy barrier and the highest exothermicity. For decomposition of all POZs, it is worth mentioning that the two routes of IM1 to IM8 and IM17 to IM17 are most likely to occur because of engendering intramolecular hydrogen bonding. The potential energy surfaces of all the POZs and Criegee intermediates involved are provided in the Figure S6. MPWB1K/6-31+g(d,p) optimized geometries for the primary ozonides, transition states and Criegee intermediates for syringol + ozone are provided in Figure S7.

Creosol contains three substituents: –OH, –OCH_3_ and –CH_3._ Like guaiacol, there are six kinds of addition pathways. Their particular thermodynamic data and the potential energy surfaces are respectively displayed in Figures S8 and S9. From Figure S4, we notice that additive site C1 is the most reactive on account of the lowest barrier. This phenomenon attributes to the electron-donating substituents, such as –OH, –OCH_3_, which is in reasonable agreement with guaiacol. The reaction of additive site C5 is the most difficult, owing to the highest barrier of 12.28 kcal/mol. From the structure of creosol, it can be seen that site C5 is the least affected by the electron donor group. The phenomenon also confirms from the reverse that the presence of these substituents do increase the activity of the near site C. Criegee intermediate IM8 is the most advantageous, and its follow-up reactions will be studied in the future. The geometries of all the primary ozonides, transition states and Criegee intermediates involved in reaction of creosol + ozone are contained in Figure S10.

## 3. Computational Details

### 3.1. Electronic Structure Calculations

All theoretical calculations were implemented by using the Gaussian 09 [37] software package (*Revision, A.02*; Gaussian, Inc., Wallingford, CT, USA). Geometry optimization of all reactants, intermediates, transition states, and products were executed using the MPWB1K method of density functional theory (DFT) in combination with a split valence polarized basis set 6-31+G(d,p). Meantime, the same method with a more flexible and accurate basis set 6-311+G(3df,2p) was employed to calculate the single-point energies of all species. Furthermore, we took advantage of intrinsic reaction coordinates (IRC) [38] for each transition state to ensure that it is indeed linked to the corresponding reactants and products.

### 3.2. Rate Constant Calculations

We derived the reaction constants including the tunneling effects from the fundamental information of ab initio calculations. All the kinetic calculations have been performed using the POLYRATE 9.7 program (University of Minnesota, Minneapolis, MN, USA) [39]. The rate constants were calculated by means of canonical variational transition state theory (CVT) with small curvature tunneling contributions (SCT) in the temperature range of 170‒370 K. For the sake of obtaining the rate constants, a series of points were selected along the minimum energy path. What′s more, they were evenly distributed on this line. The Cartesian coordinates, the gradient and the Hessian matrix were acquired by calculating the frequency of each point, and the rate coefficient was finally evaluated.

## 4. Conclusions

In conclusion, the gas-phase ozonolysis of guaiacol, syringol and creosol have been investigated theoretically in detail at the level of MPWB1K/6-311+G(3df,2p)//MPWB1K/6-31+G(d,p). Rate coefficients have been computed by using POLYRATE 9.7 based on the thermodynamic data. Some valuable conclusions are summarized as below:

(1) Six addition pathways for the initial reaction of guaiacol with ozone are considered in this work. The cycloaddition reaction of guaiacol with ozone forms primary ozonide, and then two corresponding Criegee intermediates are immediately generated by the ring-cleavage of POZ. What’s more, additive site C1 is the most active and the first channel dominates the entire reaction. Thermodynamic calculation indicates that Criegee intermediate IM8 is the most dominant.

(2) There are twelve potential secondary reactions about Criegee intermediate IM8 of guaiacol, including the reaction pathways with NO, H_2_O molecule and HOO radical, as well as its own isomerization, rearrangement and decomposition. The major degradation product of guaiacol is ascertained to be monomethyl muconate (P1), which has been detected in prior experiment. Ozonolysis of P1 generates four distinct products: Z-4-oxobut-2-enoic acid (P13), methyl 2-oxoacetate (P14), 2-oxoacetic acid (P15) and methyl (Z)-4-oxobut-2-enoate (P16).

(3) The reaction constant of the guaiacol with ozone was determined to be 1.10 × 10^−19^ cm^3^ molecule^−1^ s^−1^ at 295 K and atmospheric pressure, which is consistent with experimental results. The rate coefficients of the individual channel and the general reaction appear positive temperature dependence in the range of 170–370 K.

(4) Both the reaction mechanisms of syringol and creosol with ozone resemble that of guaiacol with ozone. As for syringol, ozone addition at site C6 is identified as the most favorable pathway and Criegee intermediate IM8 is the most stable. With regard to creosol, the route passing through transition state TS1 is the easiest to occur, and IM8 is also advantageous over others (IM7, IM9-IM18).

## Figures and Tables

**Table 1:** The overall and individual rate constants (*k_i_*/cm^−3^ molecule^−1^ s^−1^) at 1 atm and over the temperature range of 170–370 K.

T/K	*k*_1_	*k*_2_	*k*_3_	*k*_4_	*k*_5_	*k*_6_	*k*_tot_
170	4.25 × 10^−24^	2.20 × 10^−25^	7.22 × 10^−28^	7.58 × 10^−28^	7.46 × 10^−32^	1.26 × 10^−25^	4.60 × 10^−24^
195	8.01 × 10^−23^	5.57 × 10^−24^	2.50 × 10^−26^	5.27 × 10^−26^	1.33 × 10^−29^	3.60 × 10^−24^	8.93 × 10^−23^
220	7.97 × 10^−22^	7.30 × 10^−23^	3.98 × 10^−25^	1.43 × 10^−24^	7.56 × 10^−28^	4.77 × 10^−23^	9.20 × 10^−22^
245	5.10 × 10^−21^	5.67 × 10^−22^	3.68 × 10^−24^	2.04 × 10^−23^	1.93 × 10^−26^	3.91 × 10^−22^	6.08 × 10^−21^
270	2.36 × 10^−20^	3.08 × 10^−21^	2.30 × 10^−23^	1.80 × 10^−22^	2.76 × 10^−25^	2.21 × 10^−21^	2.91 × 10^−20^
295	8.62 × 10^−20^	1.28 × 10^−20^	1.08 × 10^−22^	1.12 × 10^−21^	2.57 × 10^−24^	9.50 × 10^−21^	1.10 × 10^−19^
320	2.61 × 10^−19^	4.34 × 10^−20^	4.01 × 10^−22^	5.34 × 10^−21^	1.71 × 10^−23^	3.23 × 10^−20^	3.42 × 10^−19^
345	6.85 × 10^−19^	1.25 × 10^−19^	1.25 × 10^−21^	2.05 × 10^−20^	8.82 × 10^−23^	9.55 × 10^−20^	9.27 × 10^−19^
370	1.60 × 10^−18^	3.16 × 10^−19^	3.40 × 10^−21^	6.67 × 10^−20^	3.69 × 10^−22^	2.47 × 10^−19^	2.23 × 10^−18^

**Table 2:** The branching ratios (*r*_i_) for the entrance channels at 1 atm and over the temperature range of 170–370 K.

T/K	*r*_1_	*r*_2_	*r*_3_	*r*_4_	*r*_5_	*r*_6_
170	0.9244	0.0479	0.0002	0.0002	0.0000	0.0274
195	0.8965	0.0623	0.0003	0.0006	0.0000	0.0403
220	0.8667	0.0794	0.0004	0.0016	0.0000	0.0519
245	0.8385	0.0932	0.0006	0.0034	0.0000	0.0643
270	0.8112	0.1059	0.0008	0.0062	0.0000	0.0760
295	0.7856	0.1166	0.0010	0.0102	0.0000	0.0866
320	0.7621	0.1267	0.0012	0.0156	0.0000	0.0943
345	0.7387	0.1348	0.0013	0.0221	0.0001	0.1030
370	0.7164	0.1415	0.0015	0.0299	0.0002	0.1106

## Supplementary Materials

Supplementary materials can be found at https://www.mdpi.com/1422-0067/20/18/4492/s1.
