# Supplementary material for: Mechanistic and Kinetic Investigations on the Ozonolysis of Biomass Burning Products: Guaiacol, Syringol and Creosol
Source: Int J Mol Sci. 2019 Sep 11;20(18):4492. doi: 10.3390/ijms20184492 (PMC6770092; doi:10.3390/ijms20184492)
Supplement: Supplementary file 1 [file ijms-20-04492-s001.pdf]

Supplementary Materials

Mechanistic and Kinetic Investigations on the Ozonolysis of Biomass Burning Products: Guaiacol, Syringol and Creosol

Xiaoxiao Chen[1], Yanhui Sun[1,2], Youxiao Qi[1], Lin Liu[1], Fei Xu[3] and Yan Zhao[4]*

[1] College of Environment and Safety Engineering, Qingdao University of Science and Technology, Qingdao 266042, China

[2] State Key Laboratory of Pulp and Paper Engineering, South China University of Technology, Guangzhou 510640, China

[3] Environment Research Institute, Shandong University, Qingdao 266237, China

[4] School of Life Sciences, Qufu Normal University, Qufu, 273165, China

Contents

Table S1. The energy barriers between the exo-TS and endo-TS of the six ozone-addition pathways of guaiacol, syringol and creosol at MPWB1K/6-31+G(d,p) level.

Figure S1. MPWB1K/6-31+g(d,p) optimized geometries for the primary ozonides, transition states and Criegee intermediates involved in reaction of guaiacol + ozone with main bond lengths. Bond lengths are in Å.

Figure S2. MPWB1K/6-31+g(d,p) optimized geometries for the transition states, intermediates and products with respect to the secondary reactions of IM8 with main bond lengths. Bond lengths are in Å.

Figure S3. MPWB1K/6-31+g(d,p) optimized geometries for the transition states, intermediates and products involved in further reaction of P1 with main bond lengths. Bond lengths are in Å.

Figure S4. MPWB1K/6-31+G(d,p) optimized structures of syringol and creosol.

Figure S5. The initial reaction routes of ozone with syringol, Δ*E*: potential-energy barriers, Δ*H*: heats of reaction, TS: transition state, IM: intermediate.

Figure S6. Profiles of the energy surface for the initial reaction of syringol with ozone, and the followed self-decomposition.

Figure S7. MPWB1K/6-31+g(d,p) optimized geometries for the primary ozonides, transition states and Criegee intermediates for syringol + ozone with main bond lengths. Bond lengths are in Å.

Figure S8. The initial reaction routes of ozone with creosol, Δ*E*: potential-energy barriers, Δ*H*: heats of reaction, TS: transition state, IM: intermediate.

Figure S9. Profiles of the energy surface for the initial reaction of creosol with ozone, and the followed self-decomposition.

Figure S10. MPWB1K/6-31+g(d,p) optimized geometries for the primary ozonides, transition states and Criegee intermediates involved in reaction of creosol + ozone with main bond lengths. Bond lengths are in Å.

Kinetics calculation

The rate constant for temperature T is given by:

$$k^{CVT}(T) = \min_s k^{GT}(T,s) \quad (1)$$

$$k^{GT}(T,s) = \frac{\sigma k_B T}{h} \frac{Q^{GT}(T,s)}{Q^R(T)} e^{-V_{MEP}(s)/k_B T} \quad (2)$$

where, $k^{GT}(T,s)$ is the generalized transition state theory rate constant at the dividing surface(s), T is temperature, s is the location of the generalized transition state on IRC, σ is the symmetry factor accounting for the possibility of more than one symmetry-related reaction path, k_B is Boltzmann's constant, h is Planck's constant, $Q^R(T)$ is the reactant partition function per unit volume, excluding symmetry numbers for rotation, and $Q^{GT}(T, s)$ is the partition function of a generalized transition state at s with a local zero of energy at $V_{MEP(s)}$ and with all rotational symmetry numbers set to unity. $V_{MEP(s)}$ is the potential energy at minimum energy path (MEP). To account for the tunneling effect, the CVT rate constant was multiplied by a multiplicative transmission coefficient computed with the so-called Winger correction[1].All the vibrational modes are treated as quantum-mechanical separable harmonic oscillators except for the lowest-frequency mode. The hindered rotor approximation of Truhlar and Chuang[2] is used for calculating the partition function of this lowest mode. For the SCT calculations, the effective reduced mass is obtained by a sixth-order Lagrangian interpolation.

References:

1. E.P. Wigner, On the quantum correction for thermodynamic equilibrium. Part I: Physical Chemistry. Part II: Solid State Physics. Springer, Berlin, Heidelberg, 1997, 110-120.

2. Y.Y. Chuang, D.G. Truhlar, Statistical thermodynamics of bond torsional modes. The Journal of Chemical Physics, 2000, 112, 1221-1228.

Table S1. The energy barriers between the exo-TS and endo-TS of the six ozone-addition pathways of guaiacol, syringol and creosol at MPWB1K/6-31+G(d,p) level.

Guaiacol	Δ*E* (kcal/mol)		Syringol	Δ*E*(kcal/mol)		Creosol	Δ*E*(kcal/mol)	
	exo-TS	endo-TS		exo-TS	endo-TS		exo-TS	endo-TS
TS1	12.71	7.74	TS1	10.11	6.21	TS1	11.71	6.52
TS2	12.36	8.53	TS2	14.29	10.08	TS2	11.45	7.66
TS3	13.51	10.93	TS3	11.18	9.63	TS3	11.42	8.75
TS4	10.82	9.21	TS4	10.55	8.97	TS4	9.11	7.27
TS5	15.51	13.53	TS5	14.32	9.90	TS5	14.59	12.28
TS6	10.37	8.68	TS6	9.85	5.32	TS6	9.35	7.68

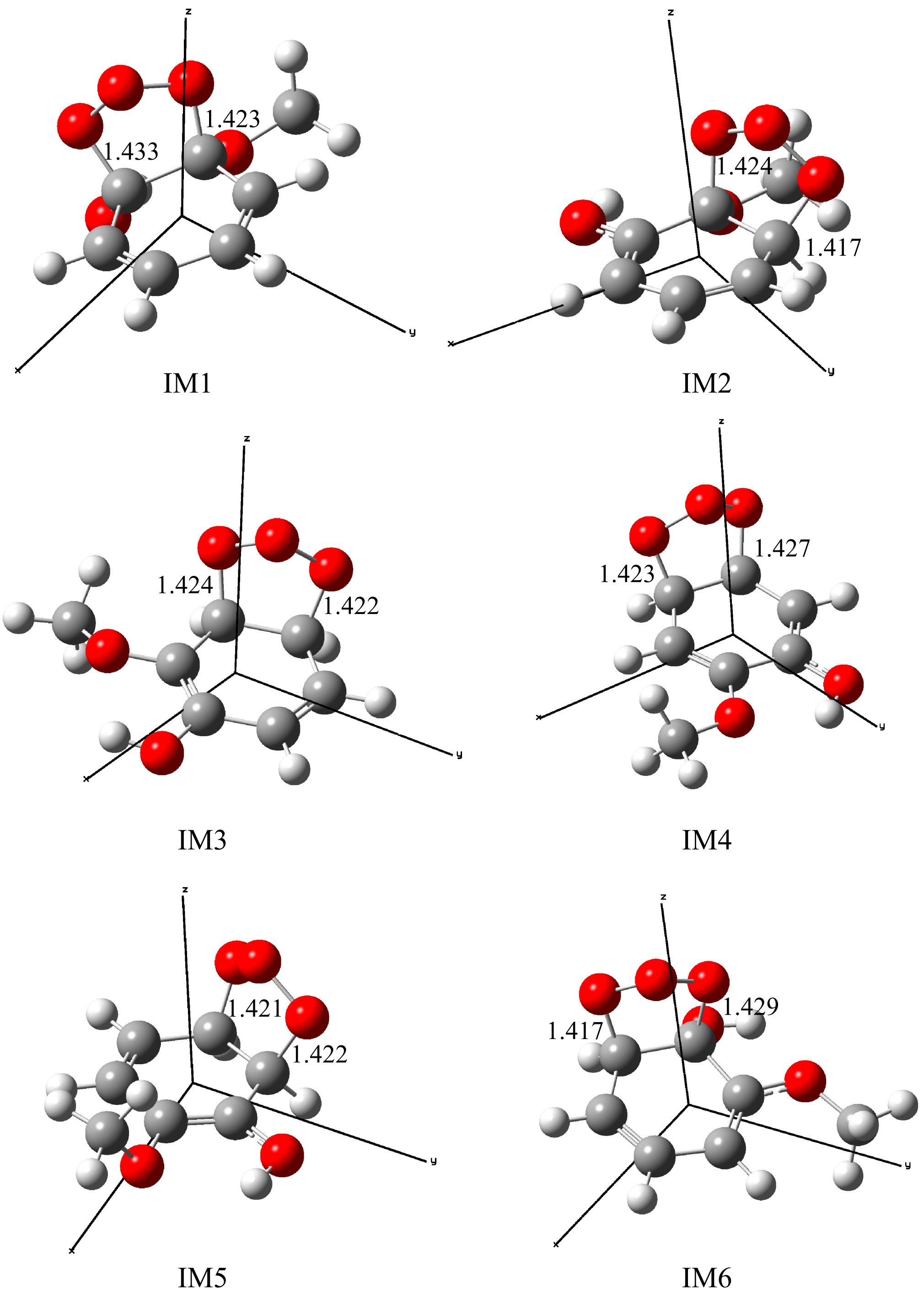

Figure S1. MPWB1K/6-31+g(d,p) optimized geometries for the primary ozonides, transition states and Criegee intermediates involved in reaction of guaiacol + ozone with main bond lengths. Bond lengths are in Å.

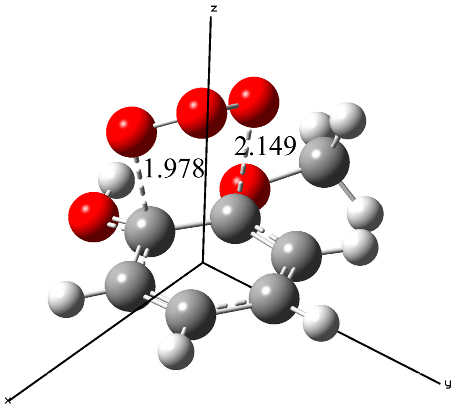

TS1

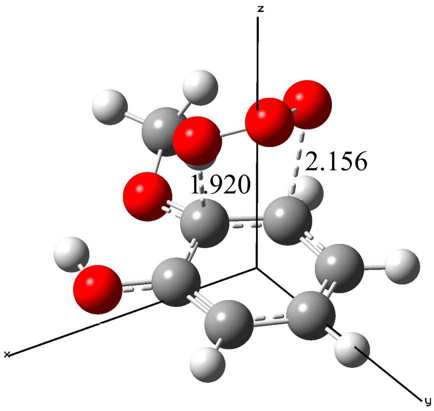

TS2

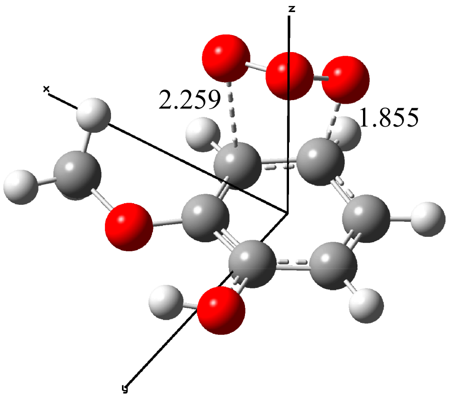

TS3

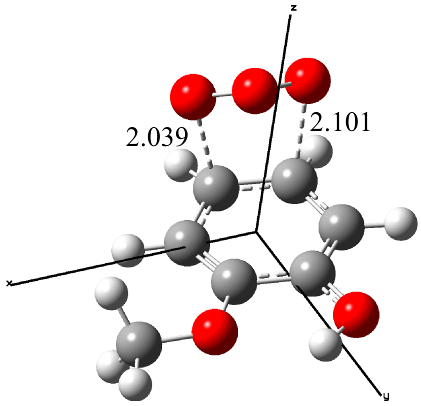

TS4

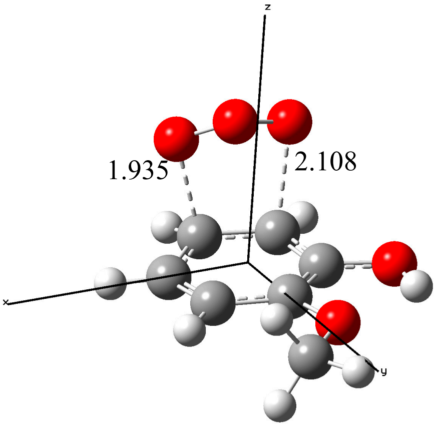

TS5

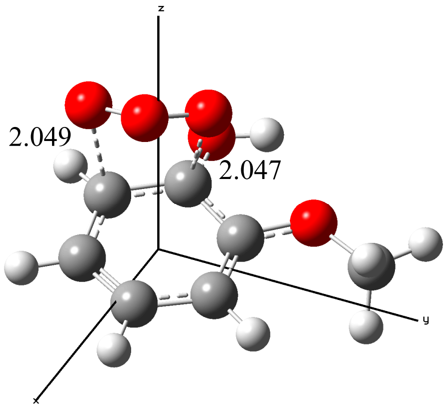

TS6

Figure S1. Continued.

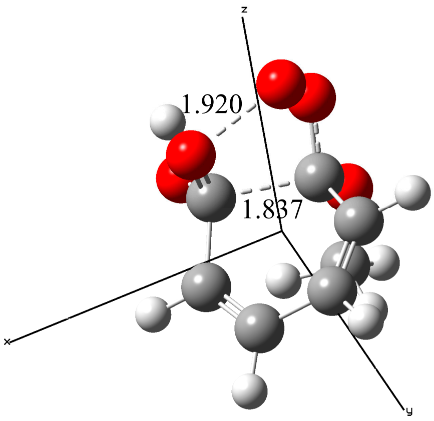

TS7

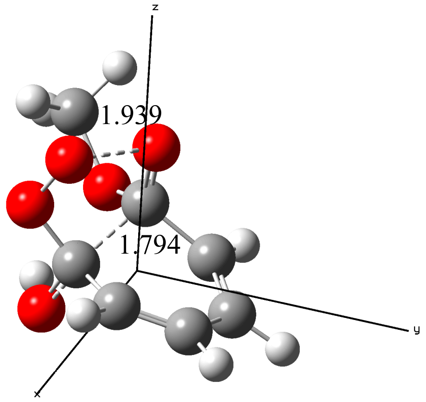

TS8

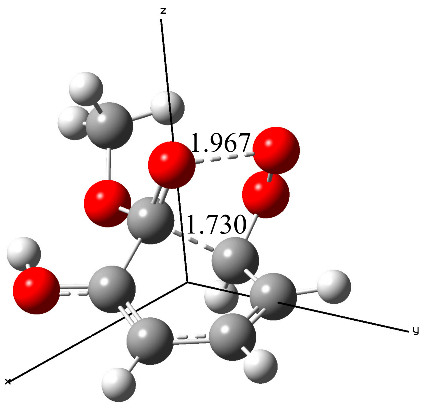

TS9

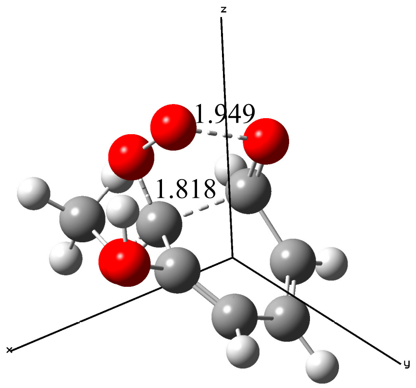

TS10

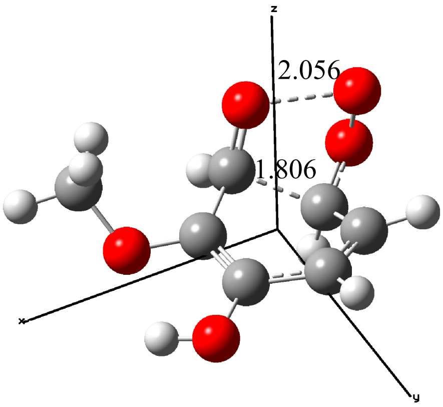

TS11

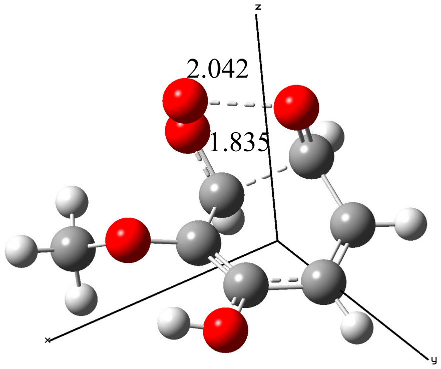

TS12

Figure S1. Continued.

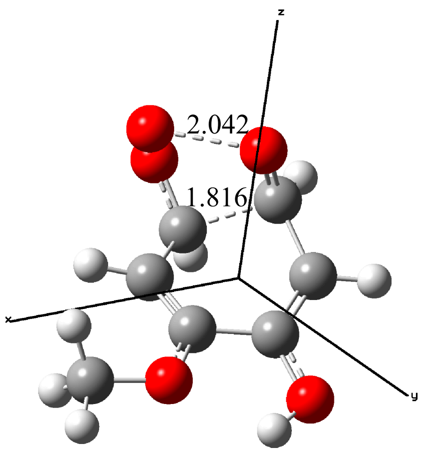

TS13

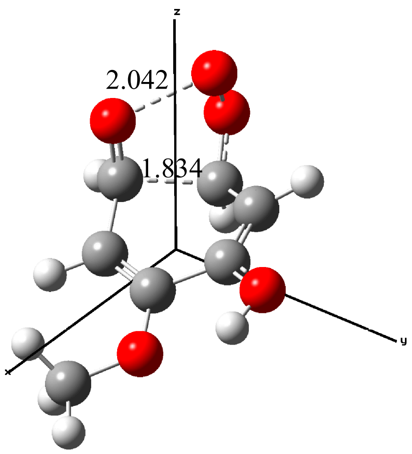

TS14

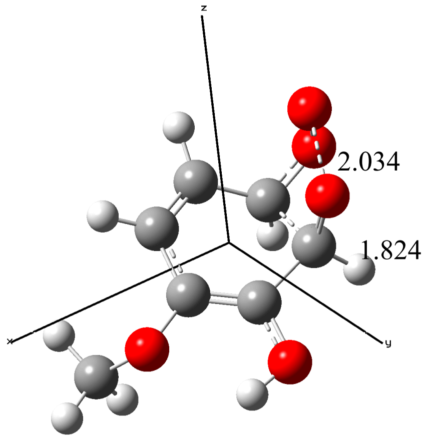

TS15

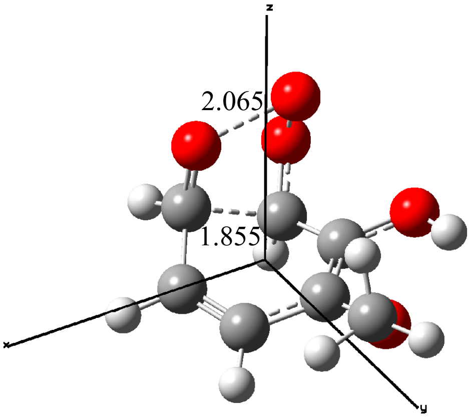

TS16

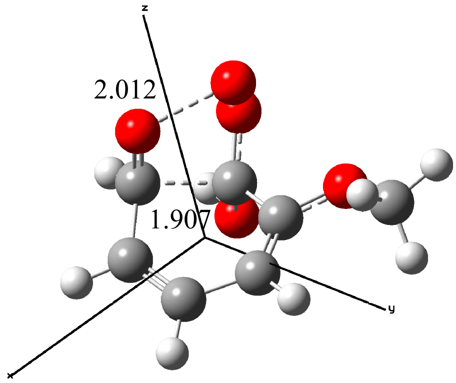

TS17

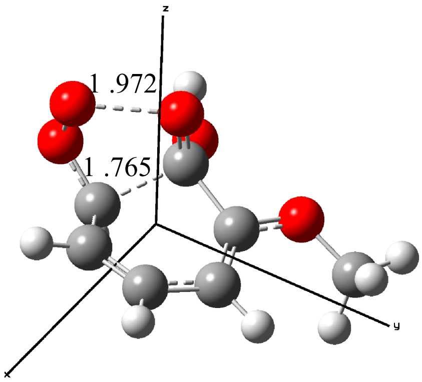

TS18

Figure S1. Continued.

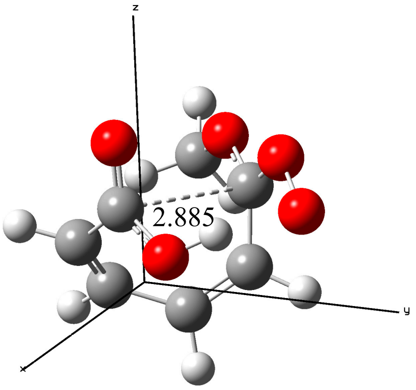

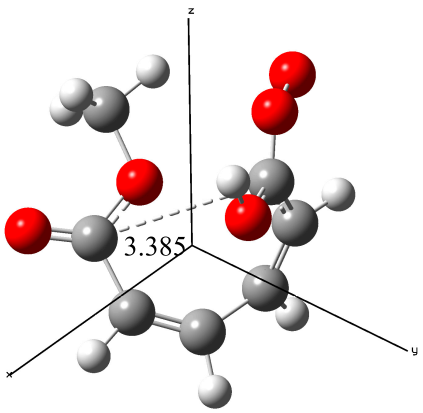

IM7

IM8

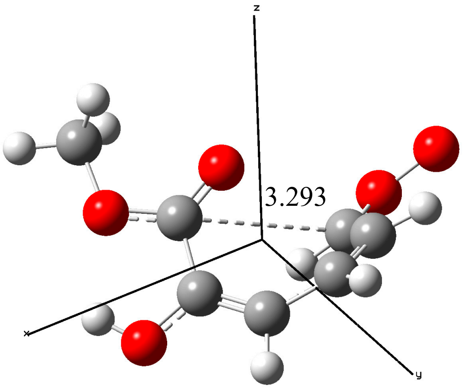

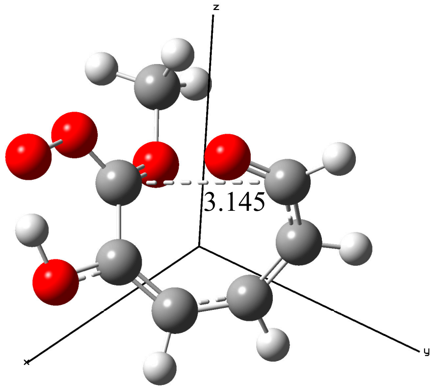

IM9

IM10

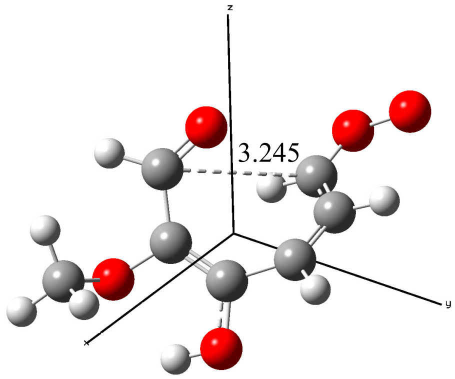

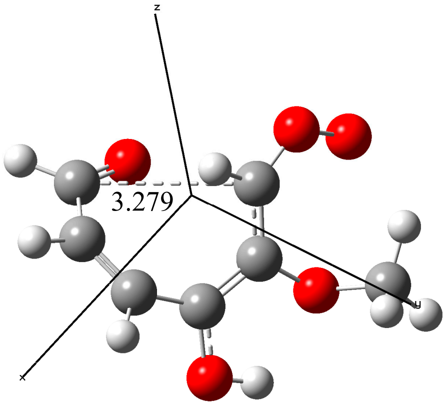

IM11

IM12

Figure S1. Continued.

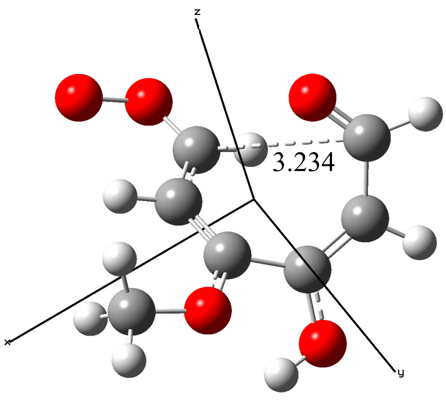

IM13

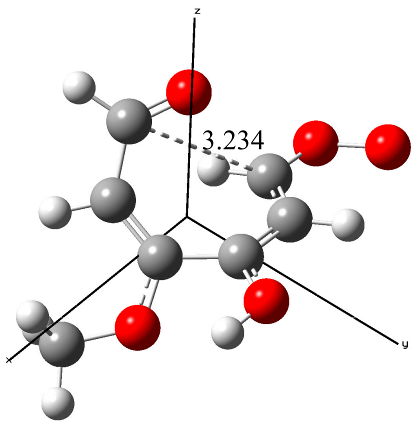

IM14

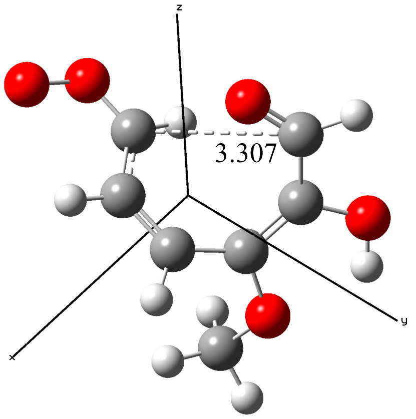

IM15

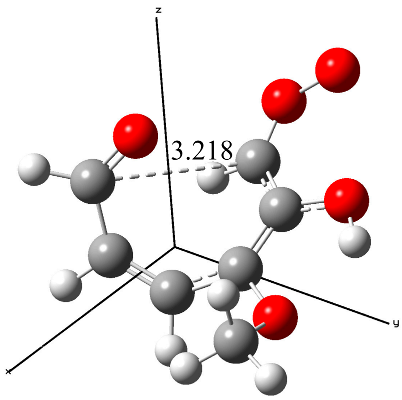

IM16

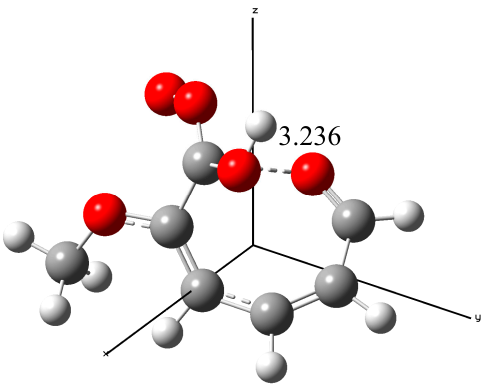

IM17

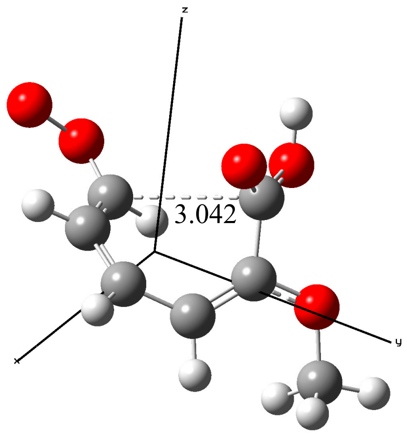

IM18

Figure S1. Continued.

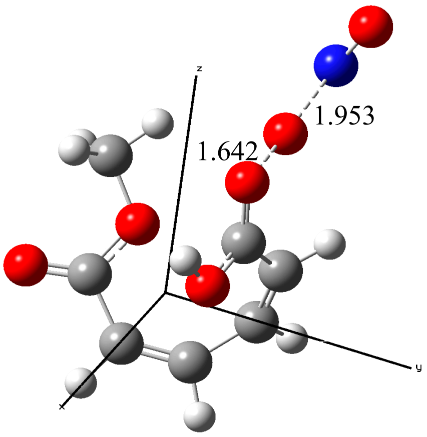

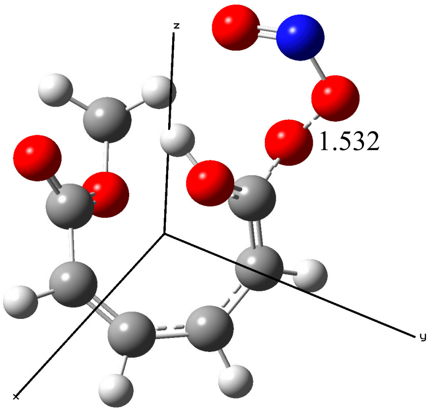

TS8-1a TS8-1b

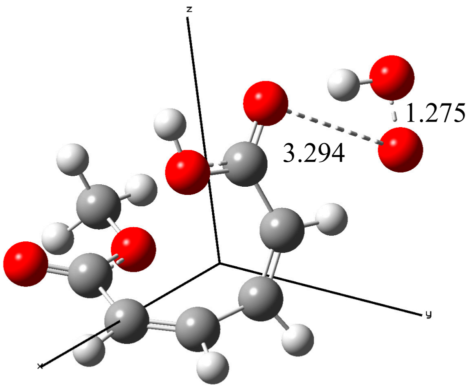

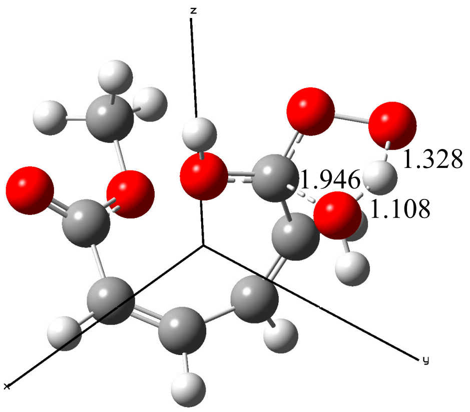

TS8-2 TS8-3

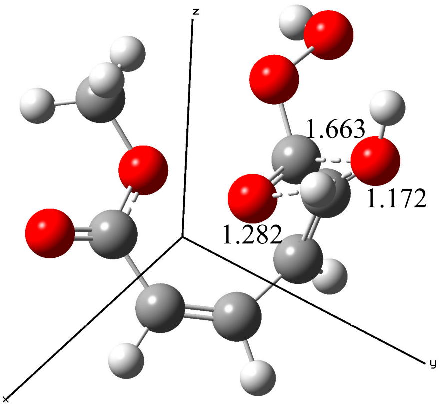

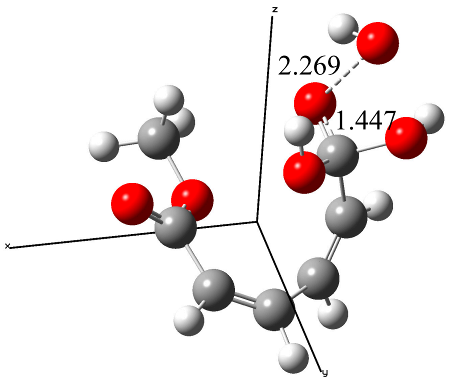

TS8-3a TS8-3b

Figure S2. MPWB1K/6-31+g(d,p) optimized geometries for the transition states, intermediates and products with respect to the secondary reactions of IM8 with main bond lengths. Bond lengths are in Å.

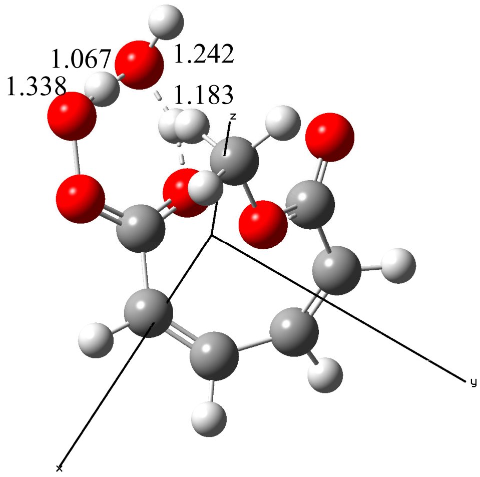

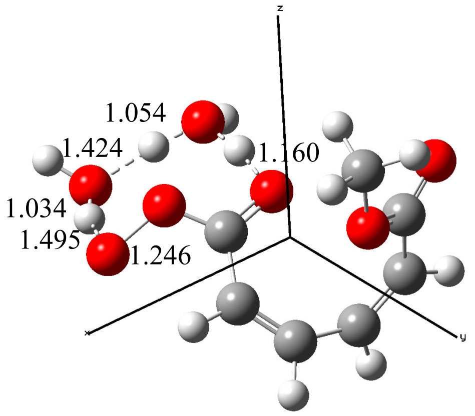

TS8-4 TS8-5

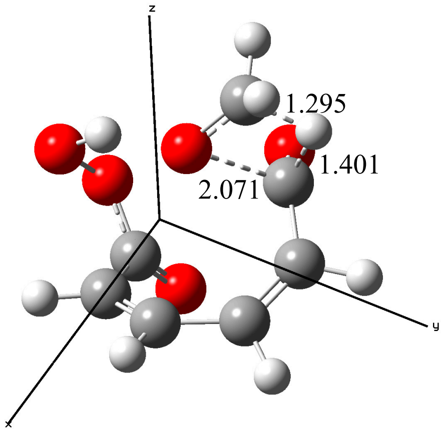

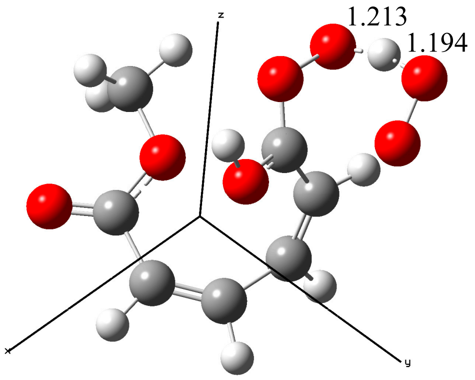

TS8-5a TS8-6

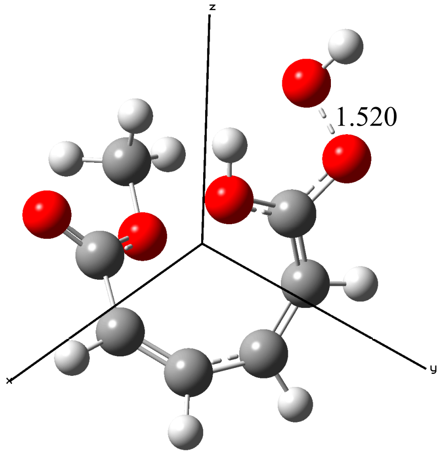

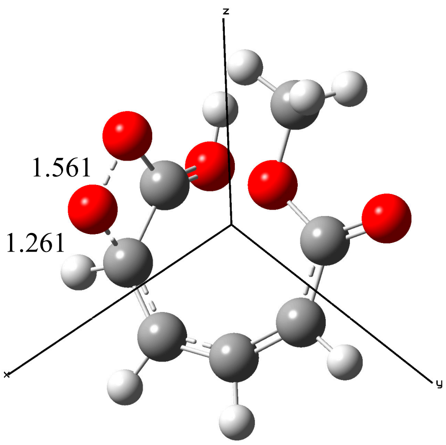

TS8-6a TS8-7

Figure S2. Continued.

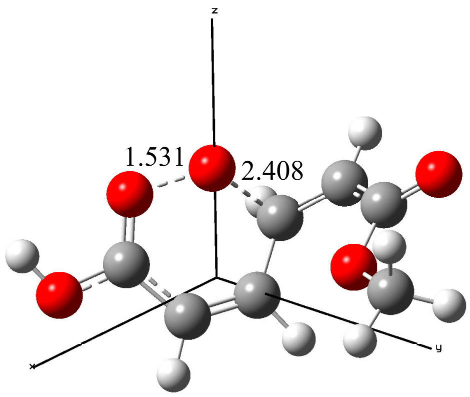

TS8-8

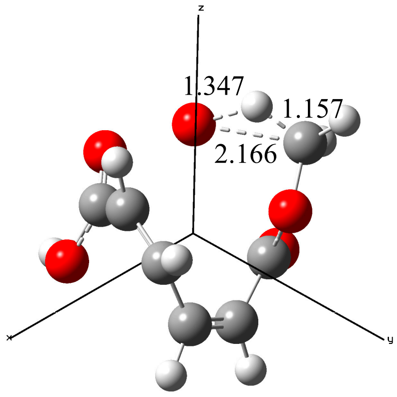

TS8-9

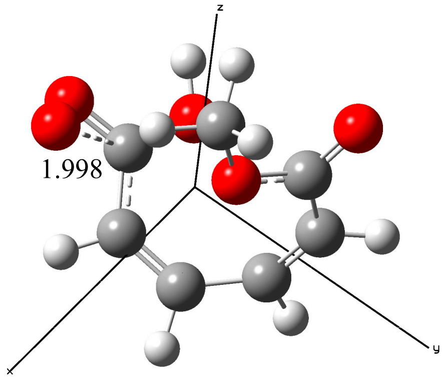

TS8-10

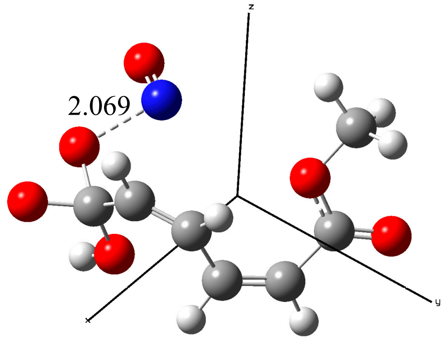

TS8-10a

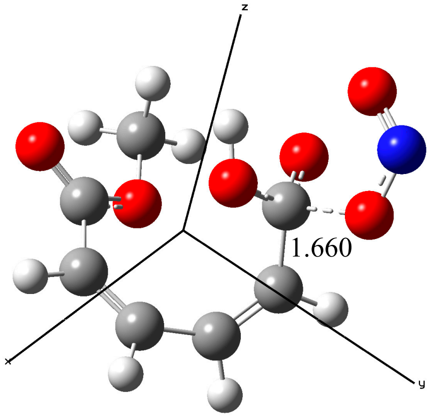

TS8-10a1

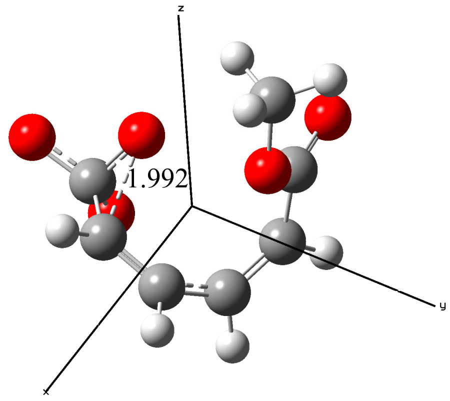

TS8-10b

Figure S2. Continued.

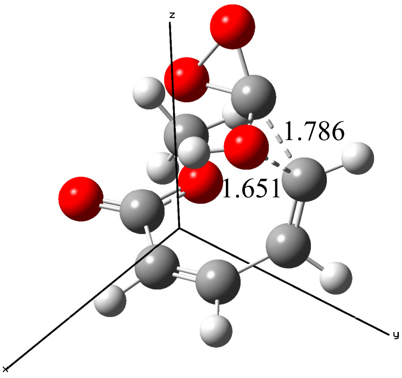

TS8-10c

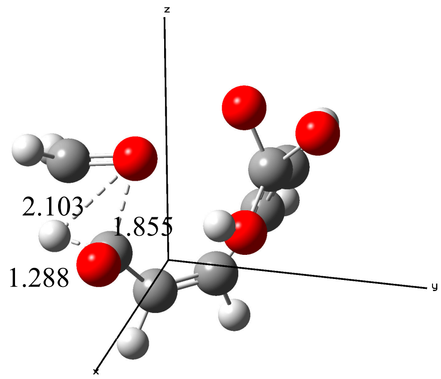

TS8-10d

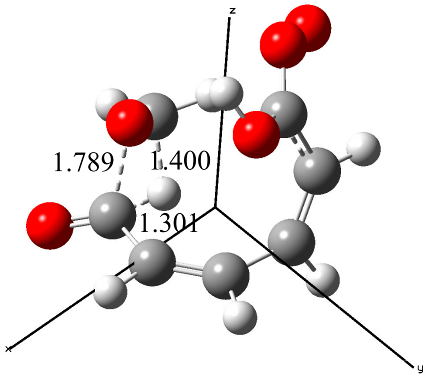

TS8-11

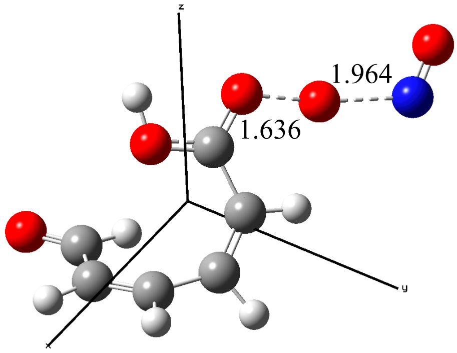

TS8-11a

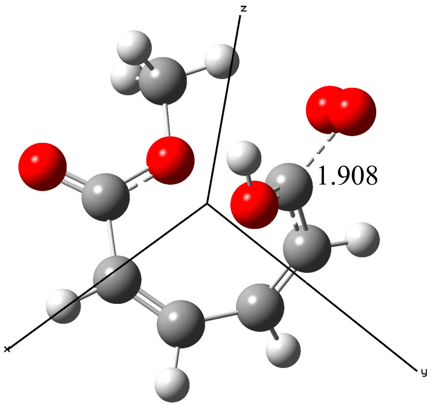

TS8-12

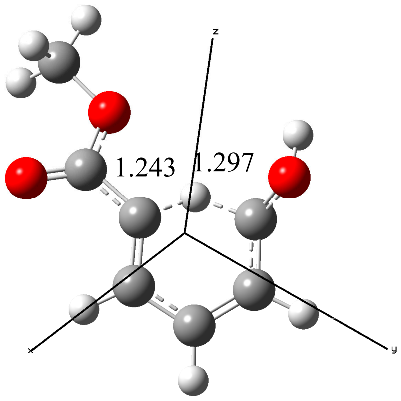

TS8-12a

Figure S2. Continued.

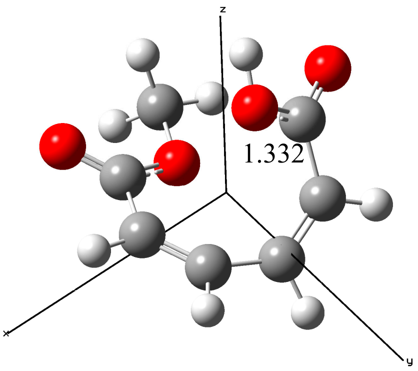

P1

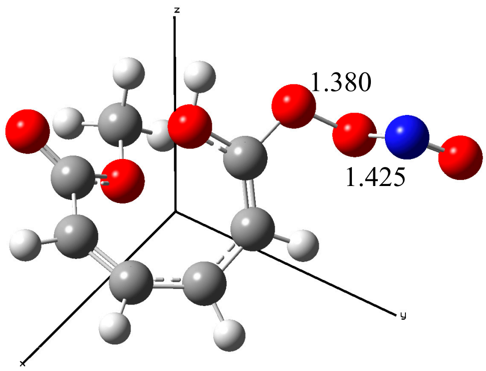

IM8-1

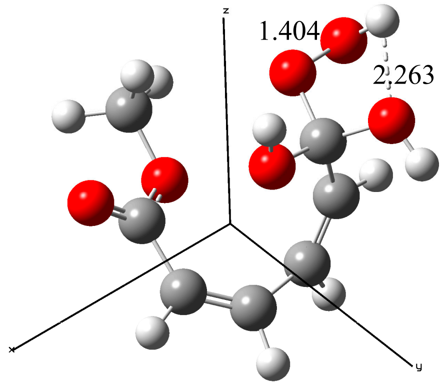

IM8-3

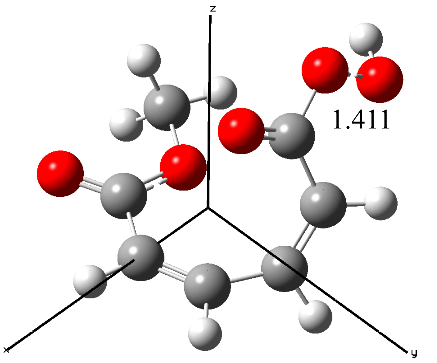

P2

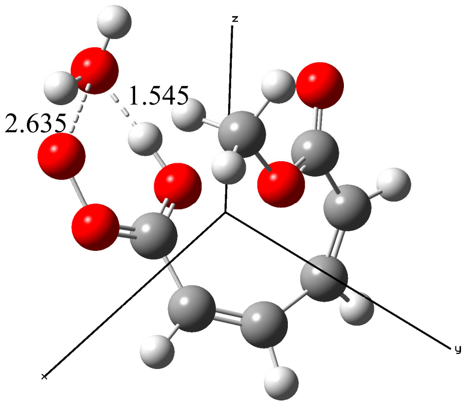

PTS8-4

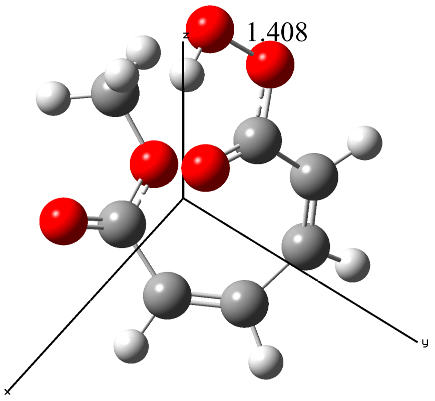

P3

Figure S2. Continued.

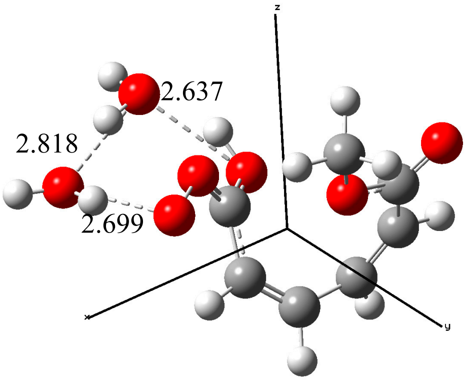

PTS8-5

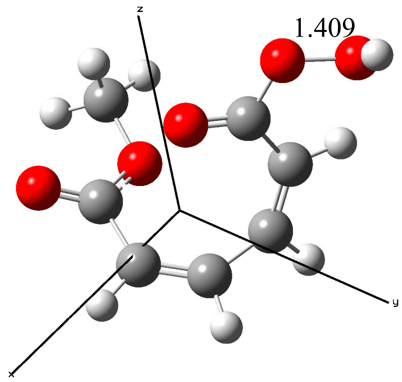

IM8-5

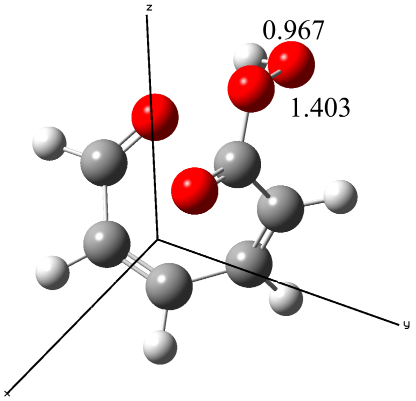

P4

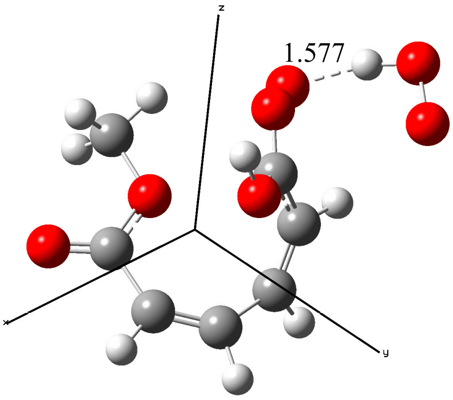

PTS8-6

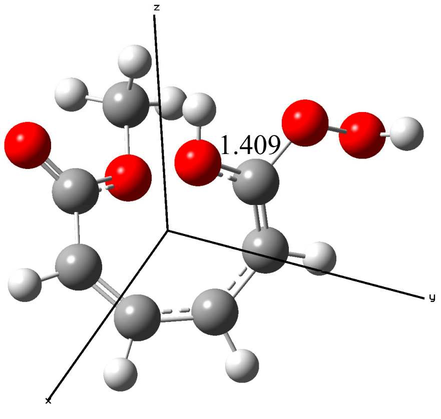

IM8-6

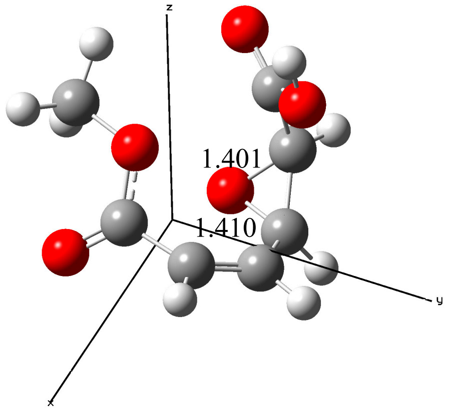

P5

Figure S2. Continued.

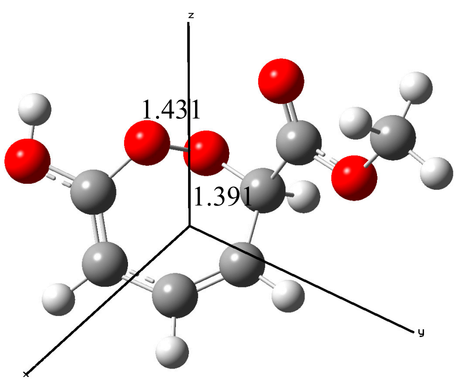

P6

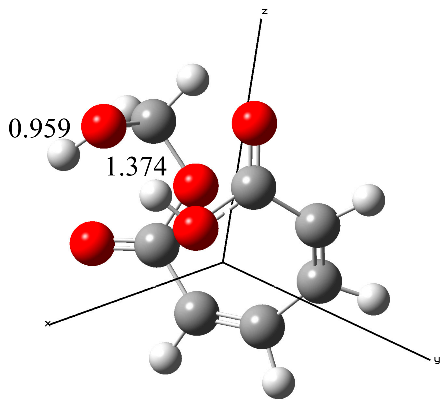

P7

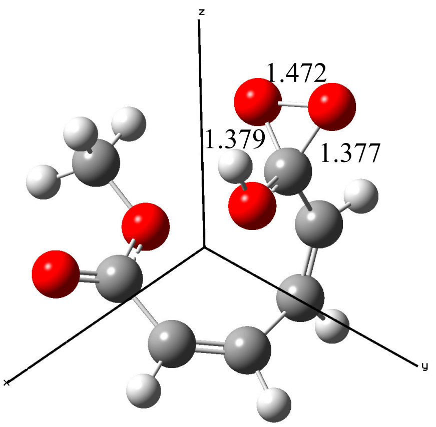

IM8-10

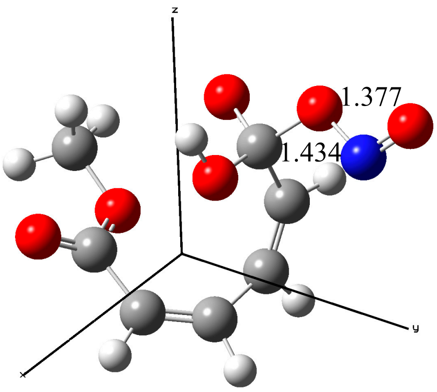

IM8-10a

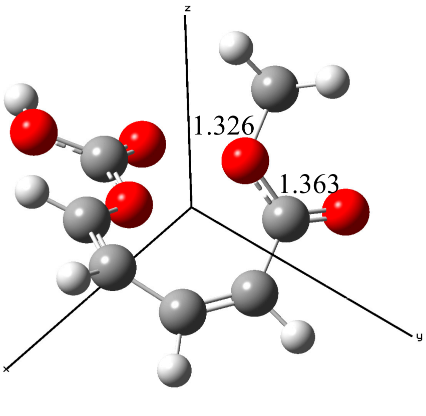

P8

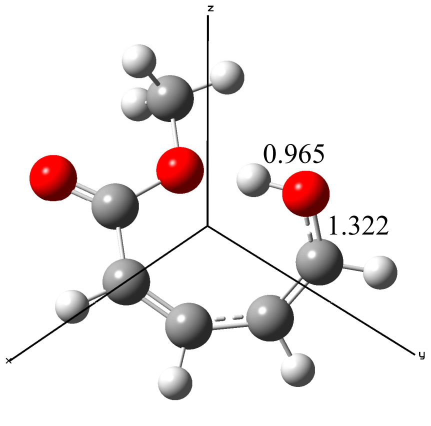

P9

Figure S2. Continued.

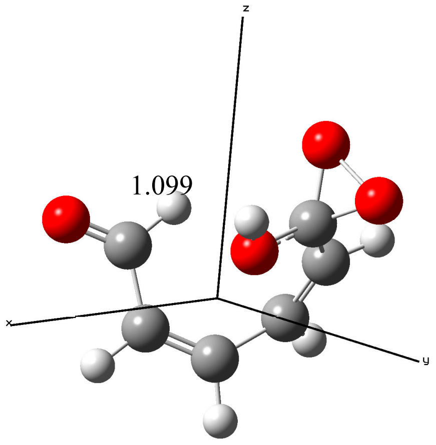

P10

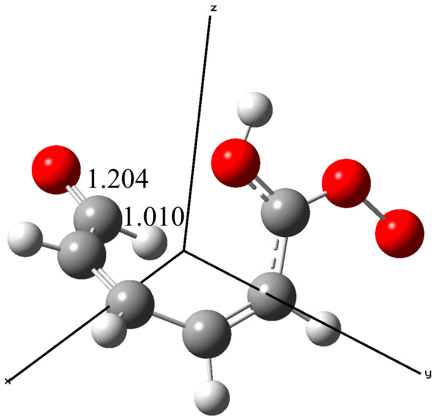

IM8-11

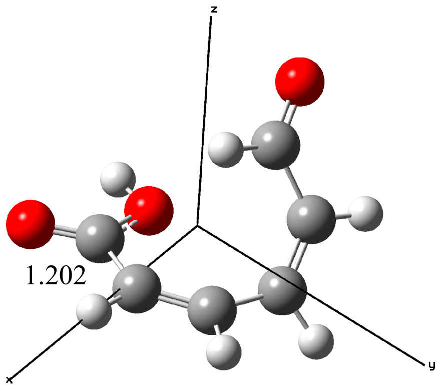

P11

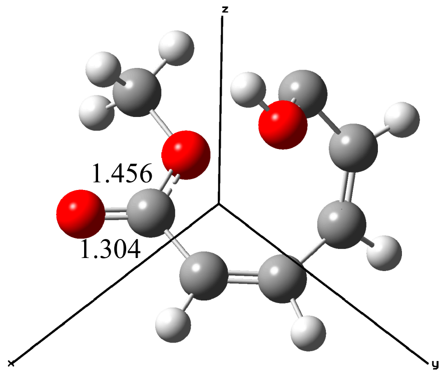

IM8-12

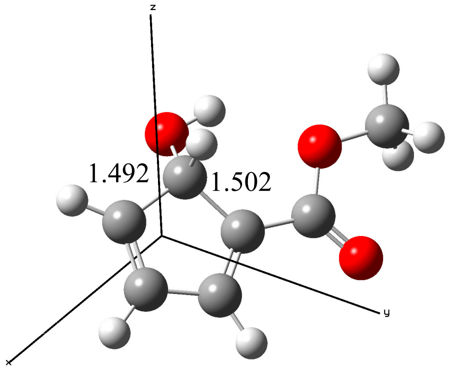

P12

Figure S2. Continued.

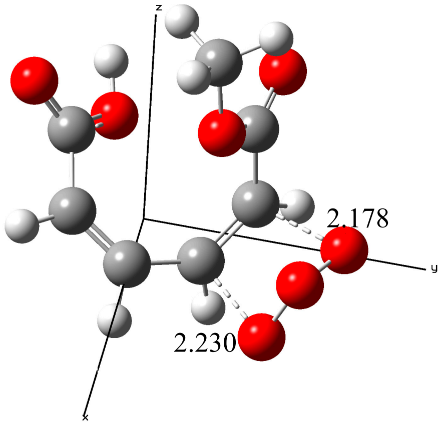

TSP1-1

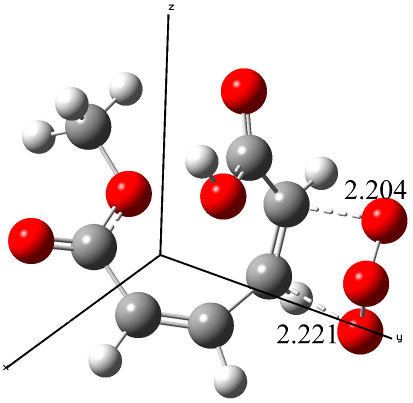

TSP1-2

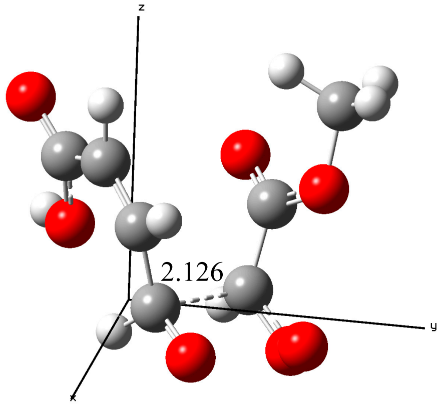

TSP1-3

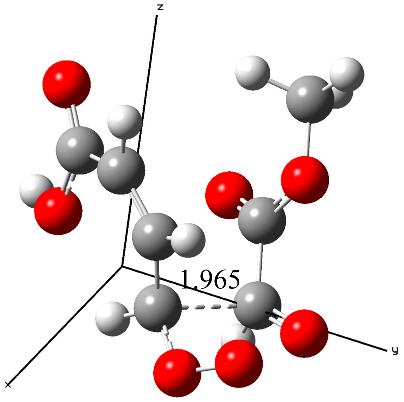

TSP1-4

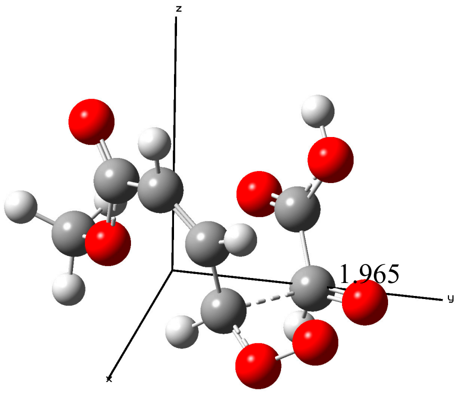

TSP1-5

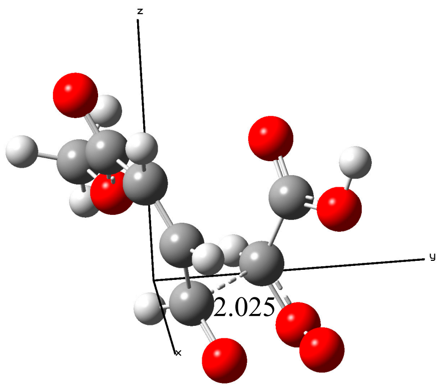

TSP1-6

Figure S3. MPWB1K/6-31+g(d,p) optimized geometries for the transition states, intermediates and products involved in further reaction of P1 with main bond lengths. Bond lengths are in Å.

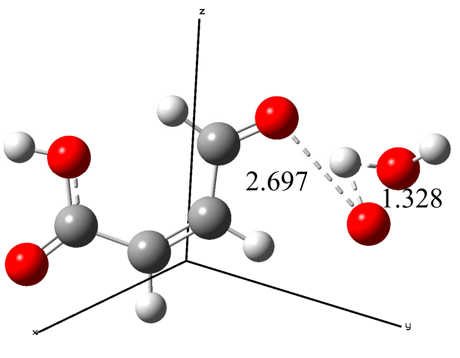

TSP1-7

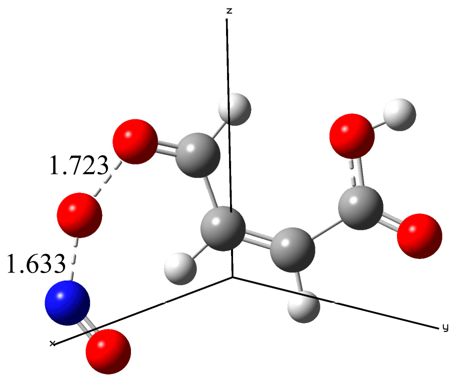

TSP1-8

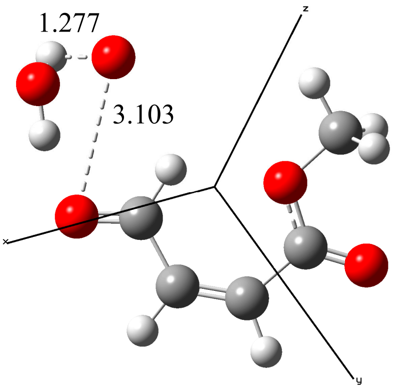

TSP1-9

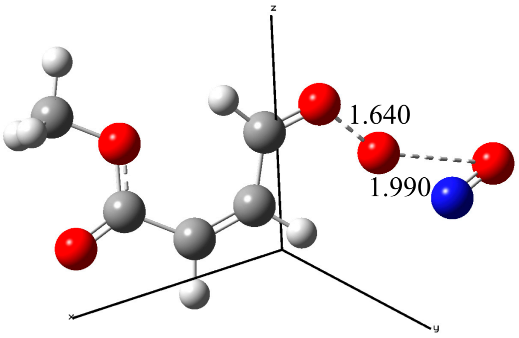

TSP1-10

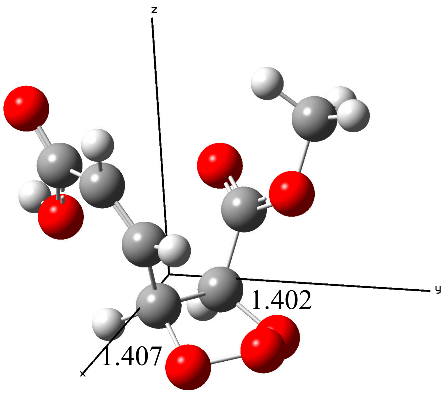

IMP1-1

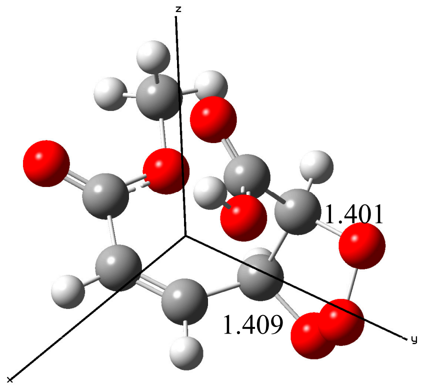

IMP1-2

Figure S3. Continued.

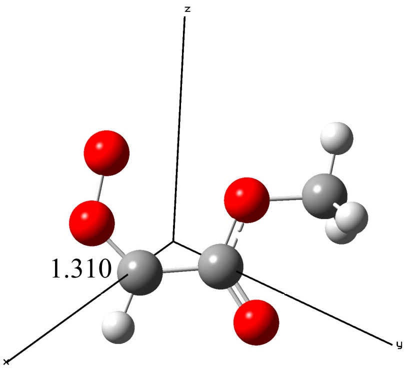

IMP1-3

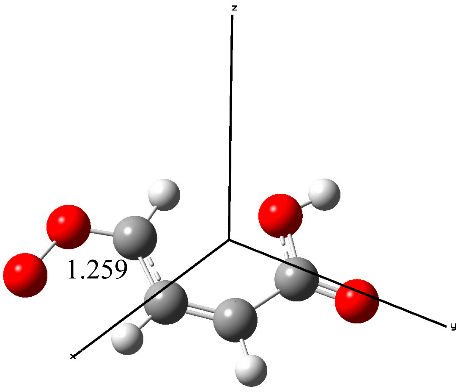

IMP1-4

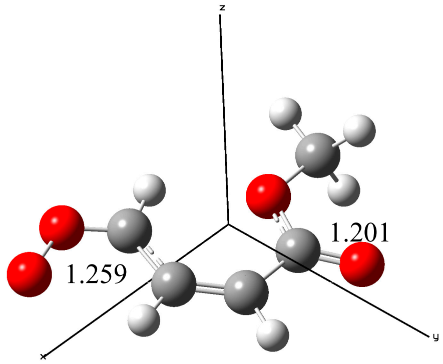

IMP1-5

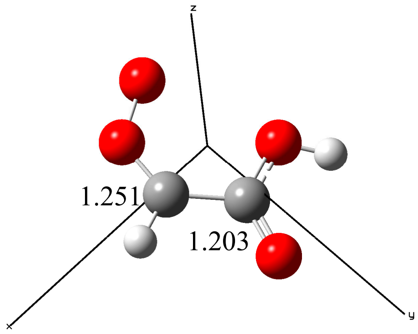

IMP1-6

Figure S3. Continued.

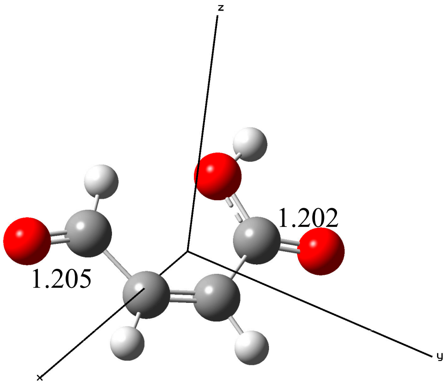

P13

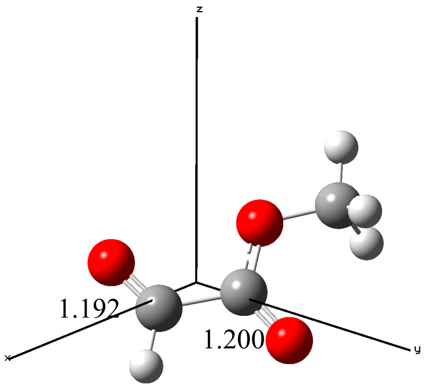

P14

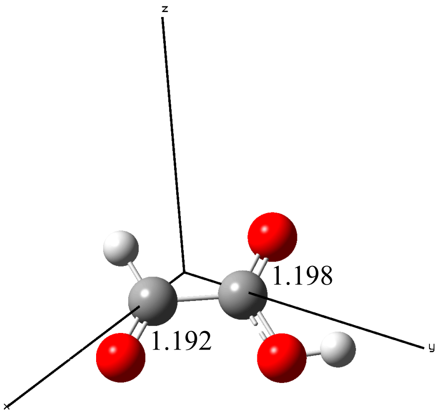

P15

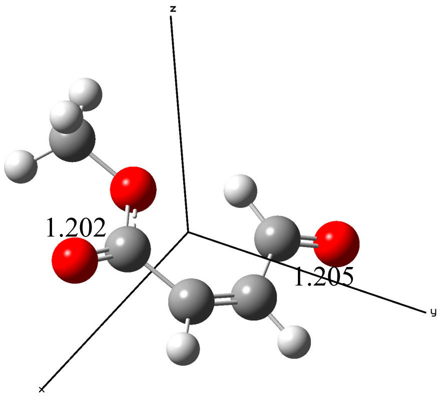

P16

Figure S3. Continued.

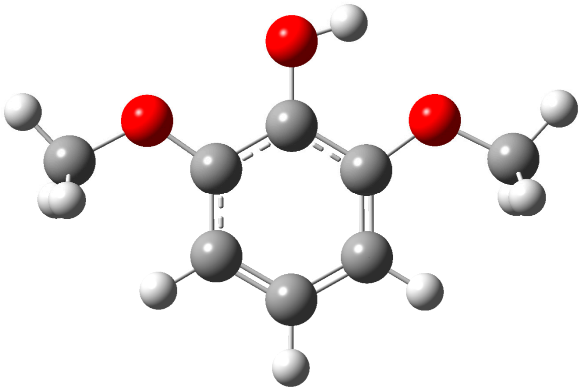

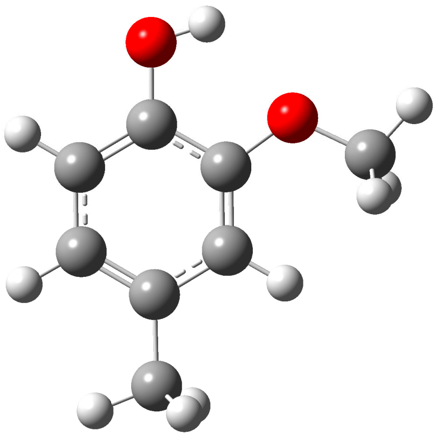

syringol **creosol**

Figure S4. MPWB1K/6-31+G(d,p) optimized structures of syringol and creosol.

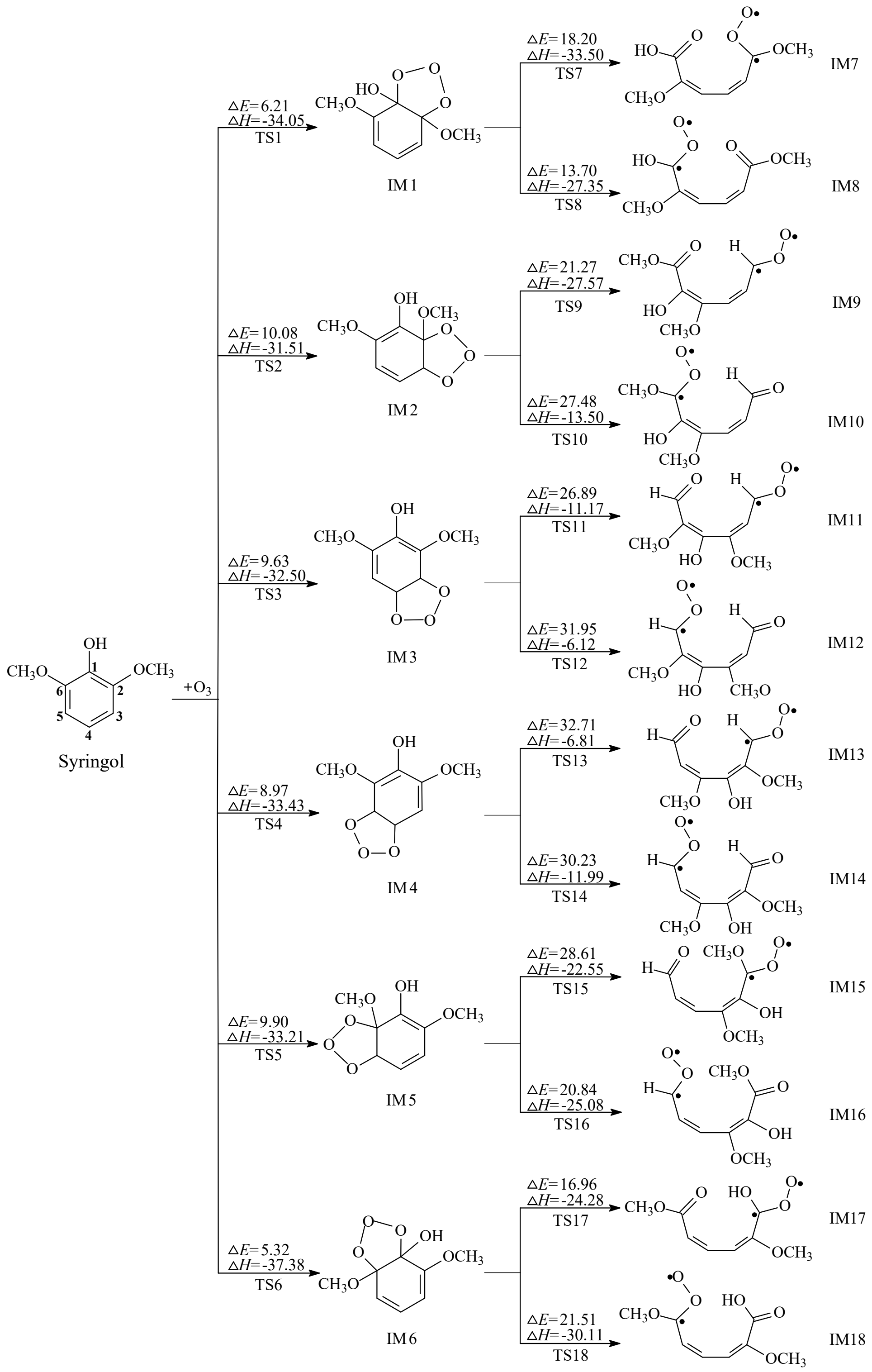

Figure S5. The initial reaction routes of ozone with syringol, Δ*E*: potential-energy barriers, Δ*H*: heats of reaction, TS: transition state, IM: intermediate.

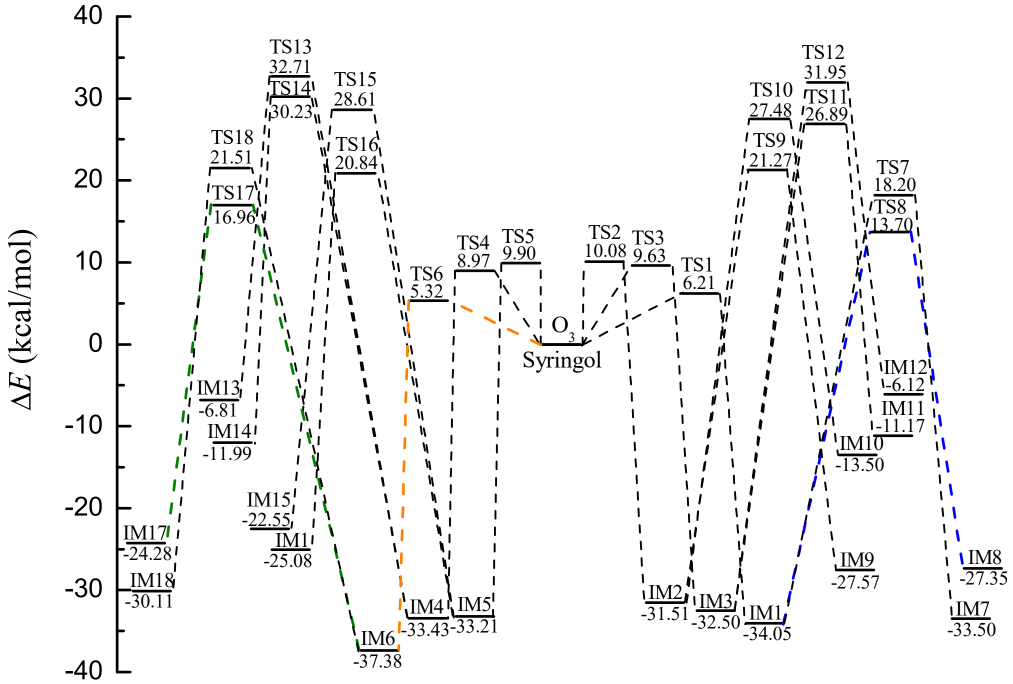

Figure S6. Profiles of the energy surface for the initial reaction of syringol with ozone, and the followed self-decomposition.

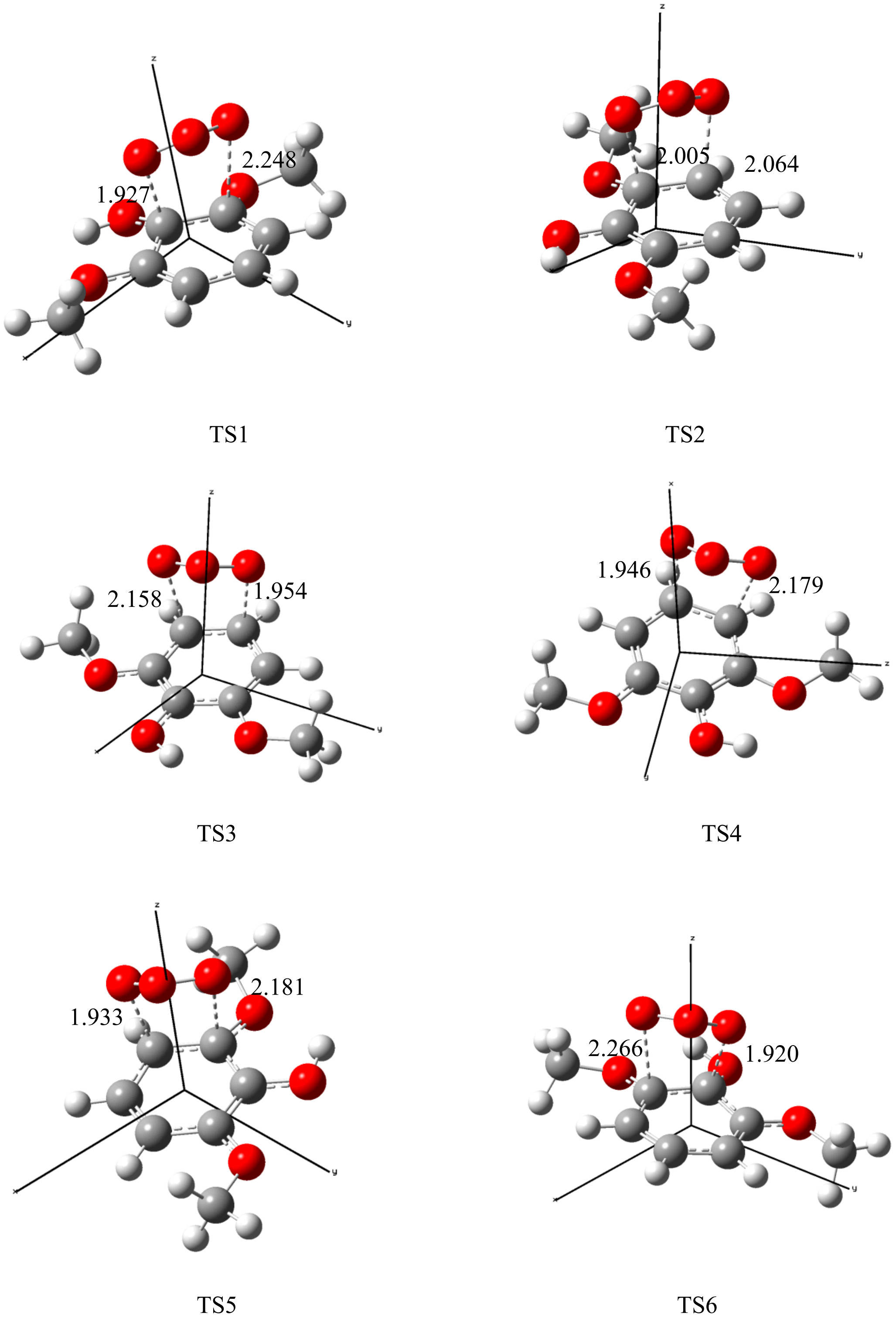

Figure S7. MPWB1K/6-31+g(d,p) optimized geometries for the primary ozonides, transition states and Criegee intermediates for syringol + ozone with main bond lengths. Bond lengths are in Å.

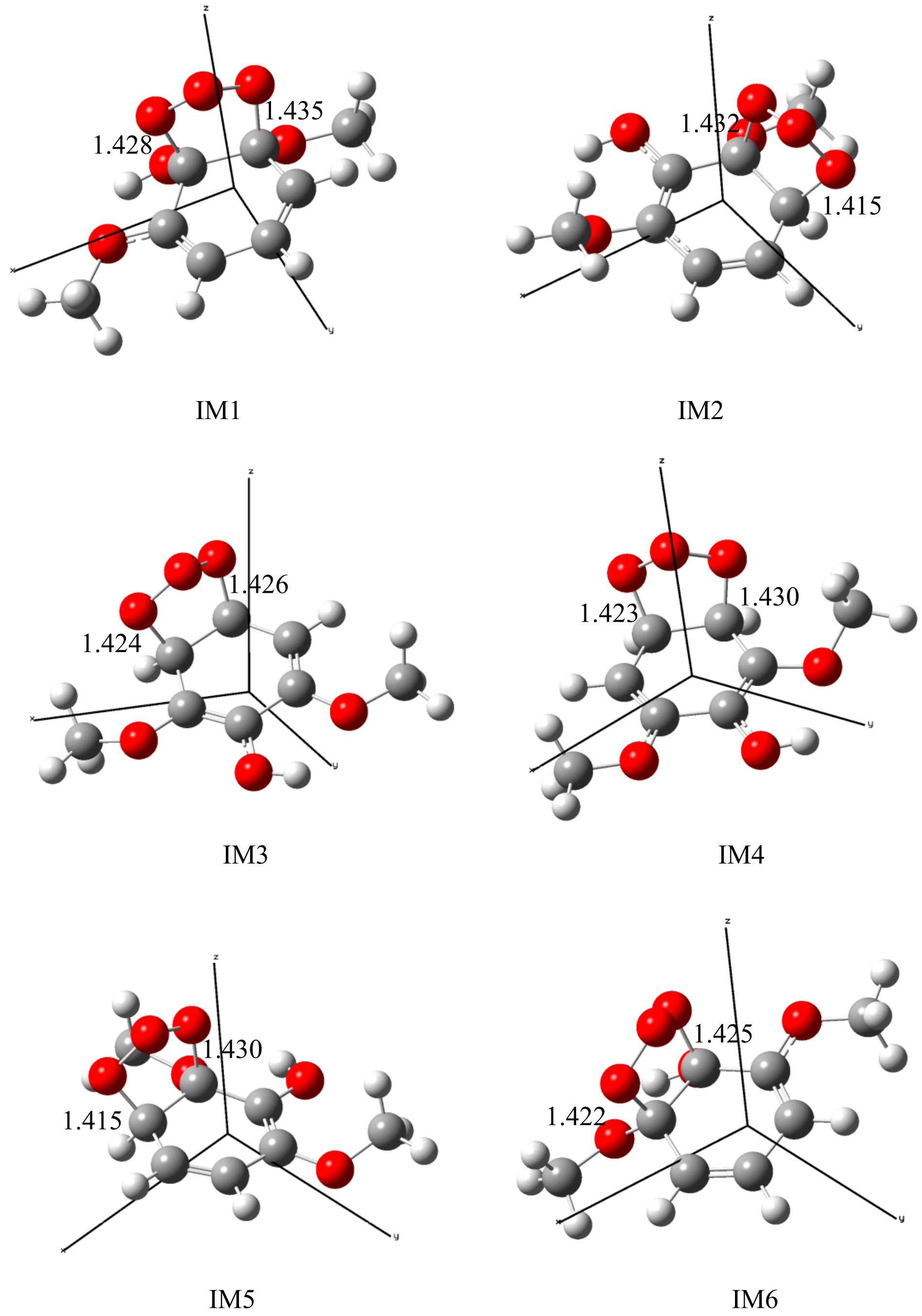

Figure S7. Continued.

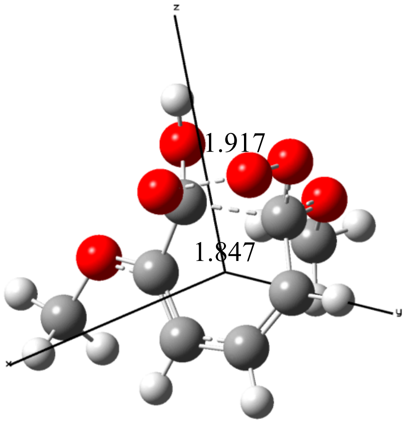

TS7

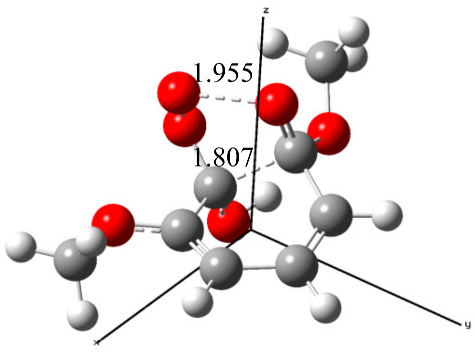

TS8

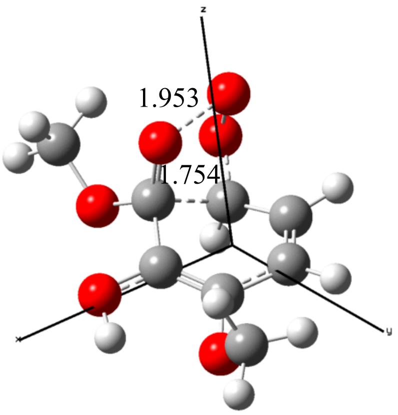

TS9

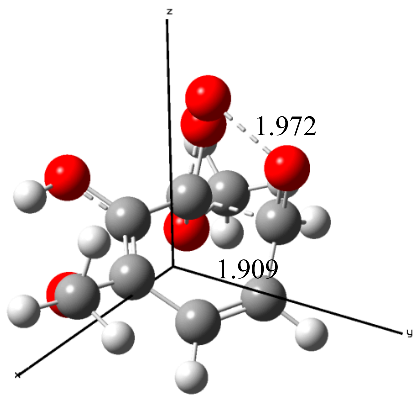

TS10

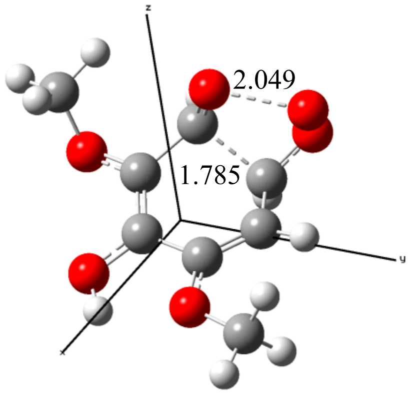

TS11

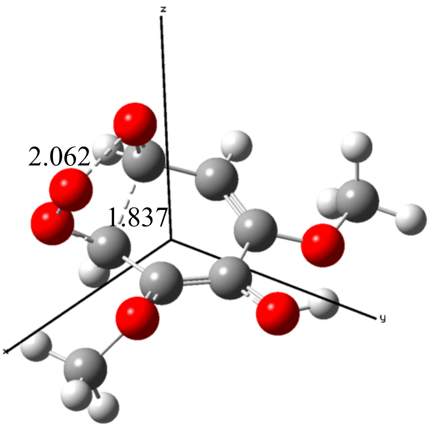

TS12

Figure S7. Continued.

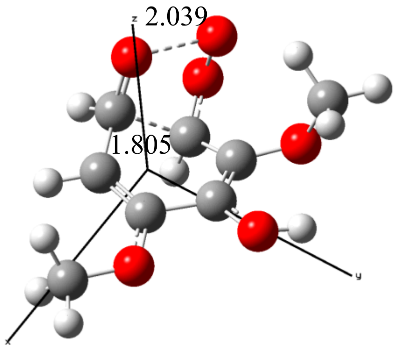

TS13

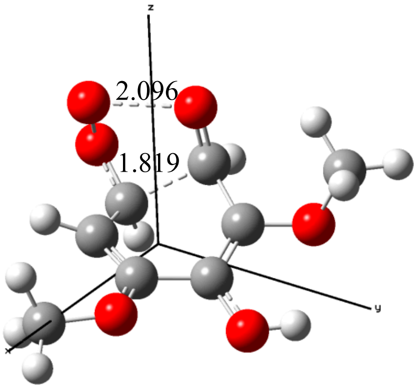

TS14

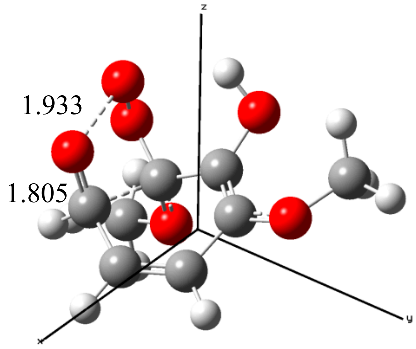

TS15

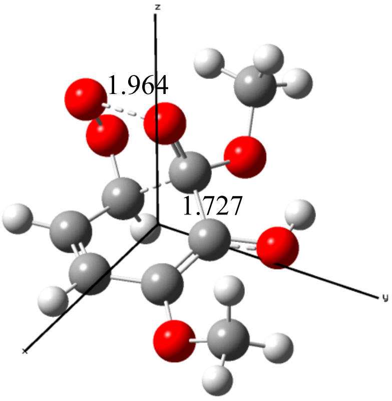

TS16

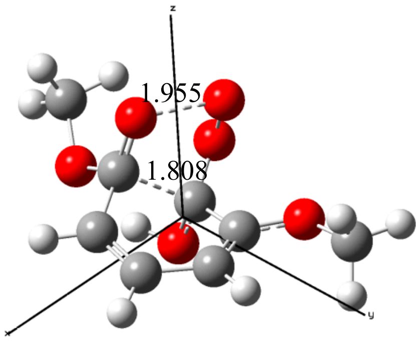

TS17

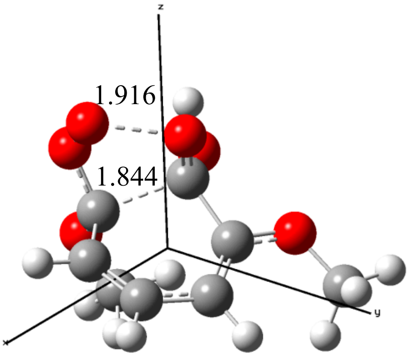

TS18

Figure S7. Continued.

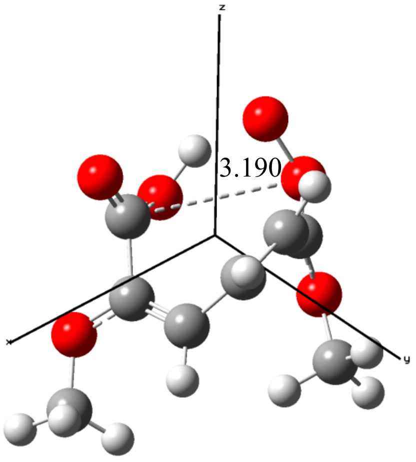

IM7

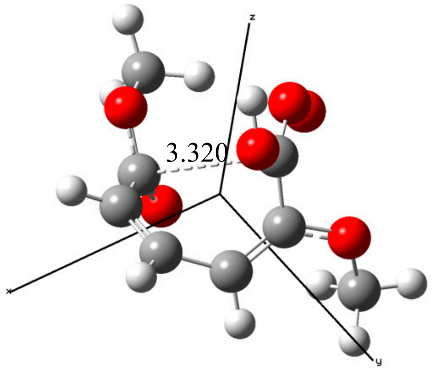

IM8

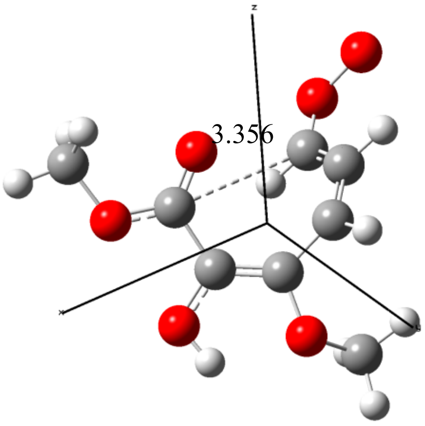

IM9

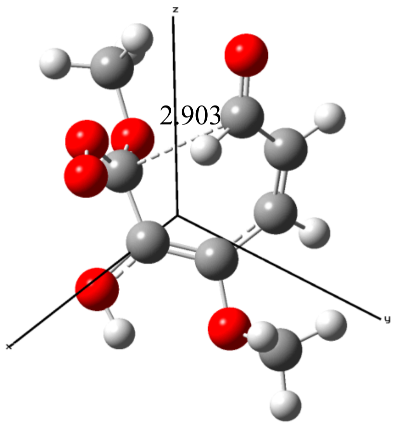

IM10

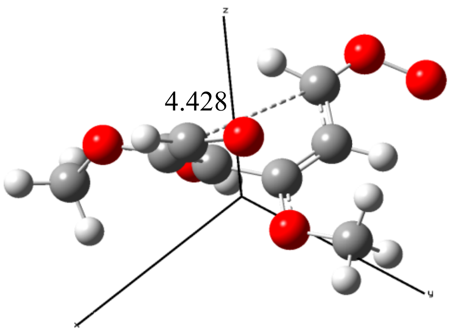

IM11

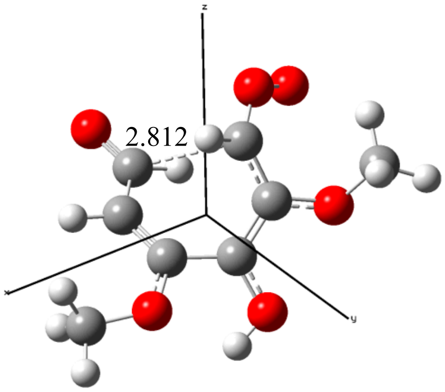

IM12

Figure S7. Continued.

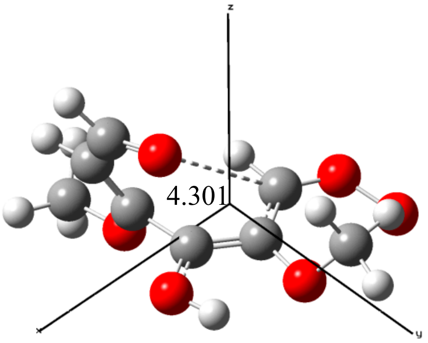

IM13

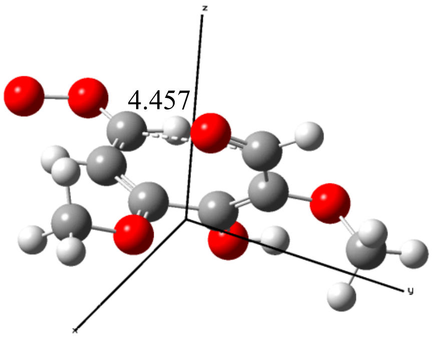

IM14

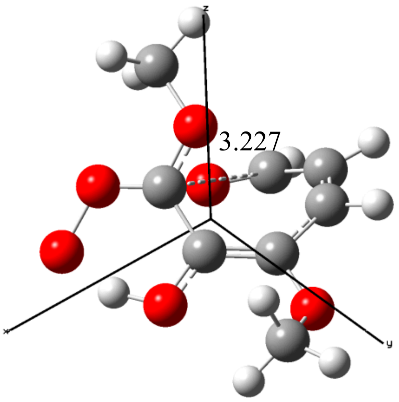

IM15

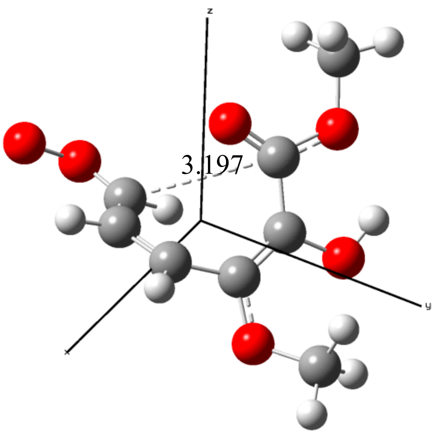

IM16

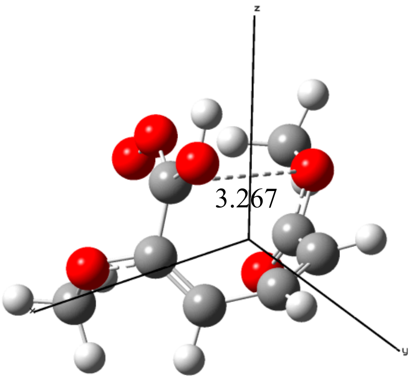

IM17

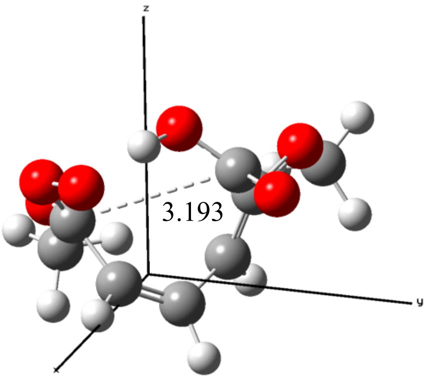

IM18

Figure S7. Continued.

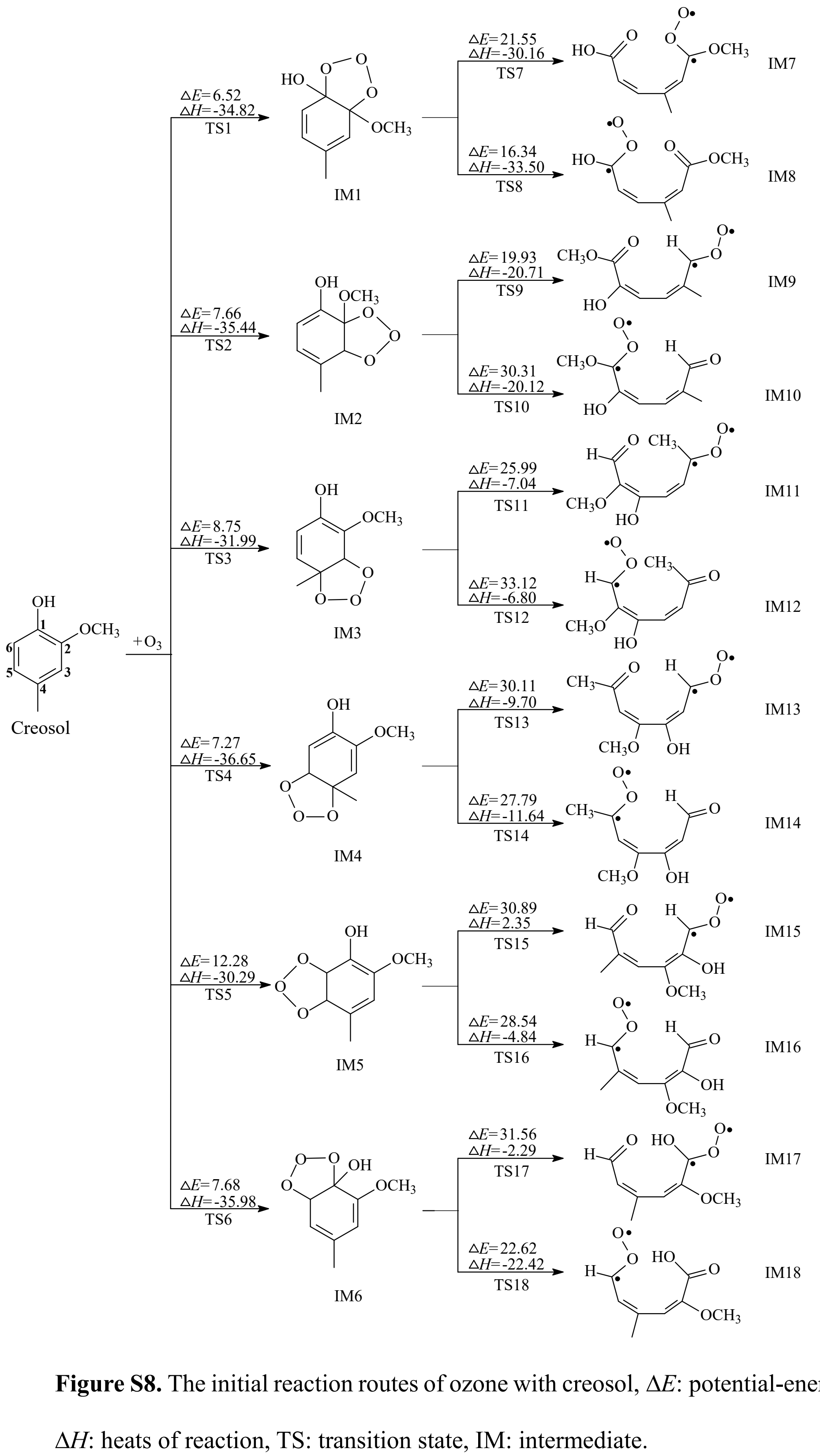

Figure S8. The initial reaction routes of ozone with creosol, Δ*E*: potential-energy barriers, Δ*H*: heats of reaction, TS: transition state, IM: intermediate.

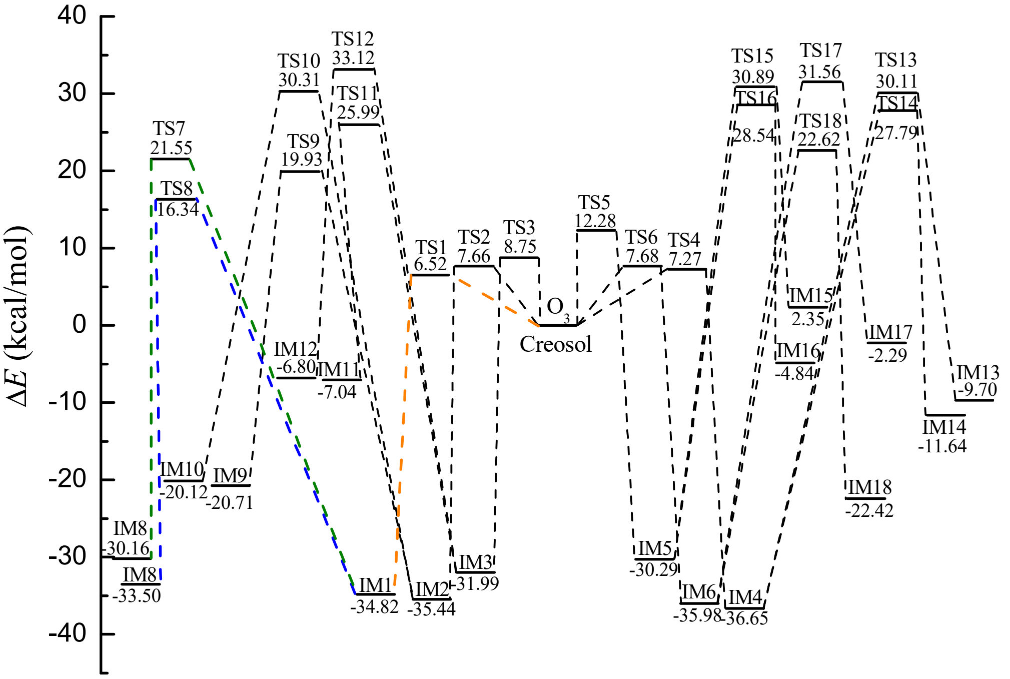

Figure S9. Profiles of the energy surface for the initial reaction of creosol with ozone, and the followed self-decomposition.

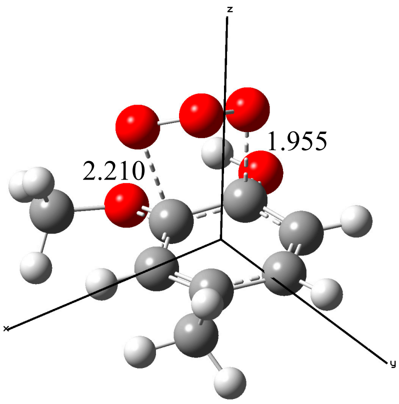

TS1

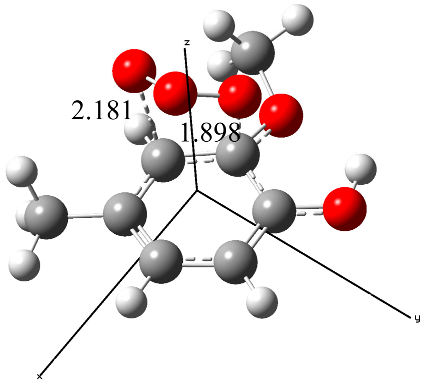

TS2

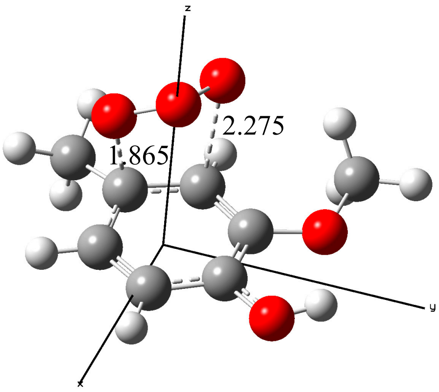

TS3

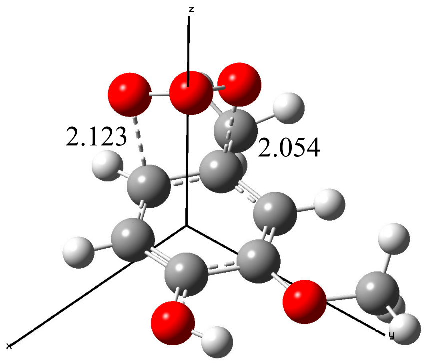

TS4

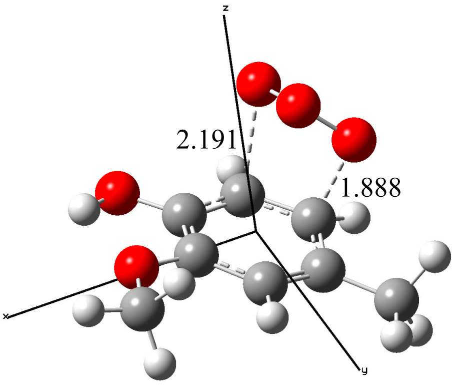

TS5

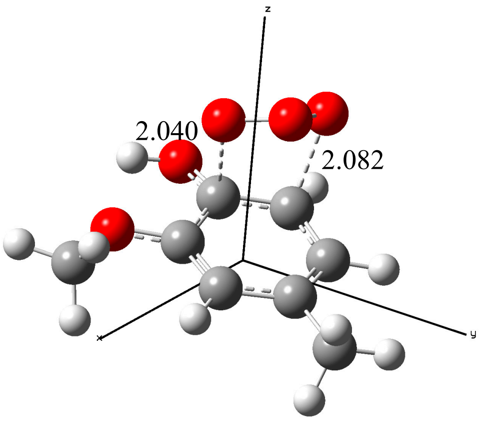

TS6

Figure S10. MPWB1K/6-31+g(d,p) optimized geometries for the primary ozonides, transition states and Criegee intermediates involved in reaction of creosol + ozone with main bond lengths. Bond lengths are in Å.

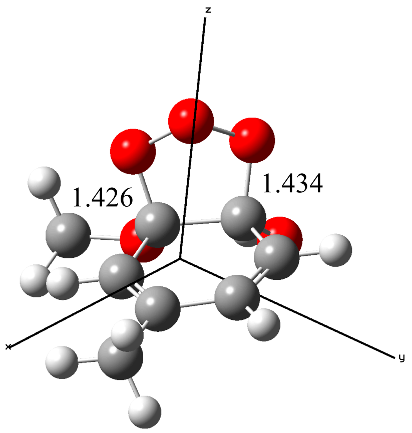

IM1

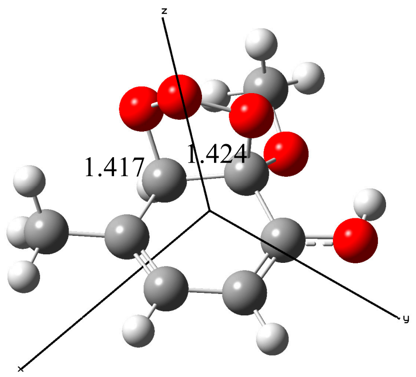

IM2

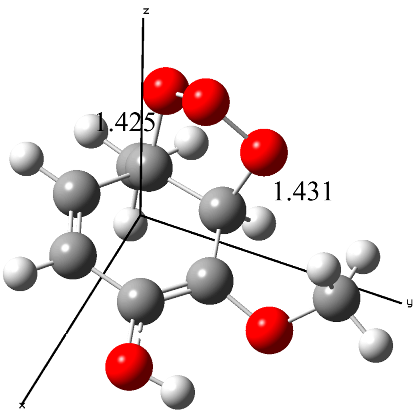

IM3

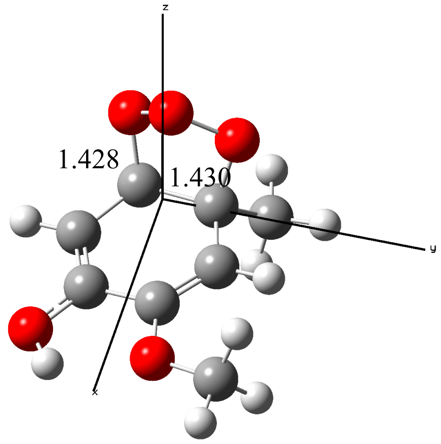

IM4

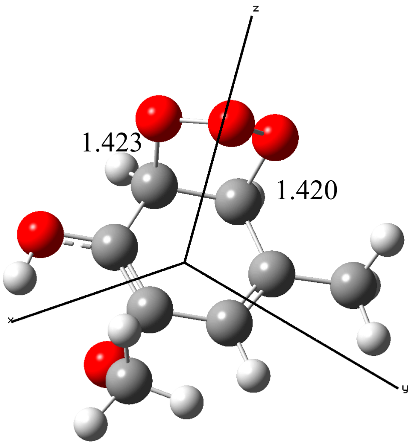

IM5

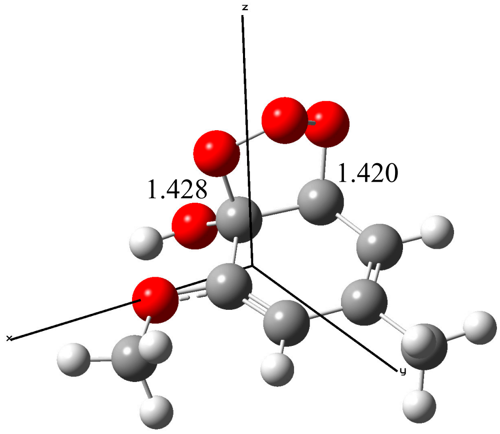

IM6

Figure S10. Continued.

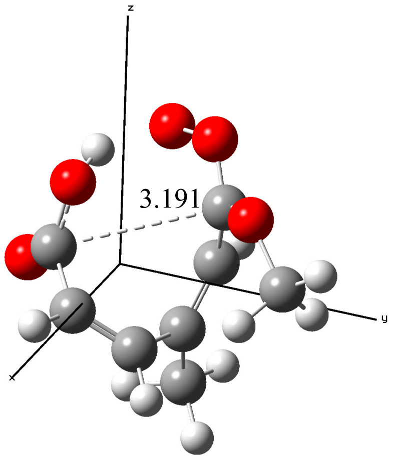

IM7

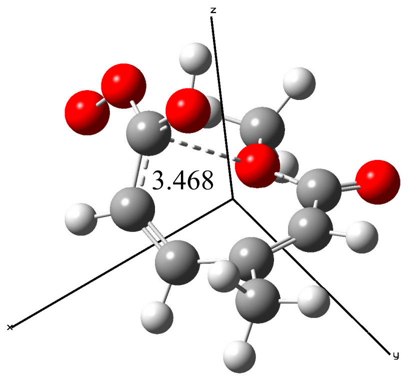

IM8

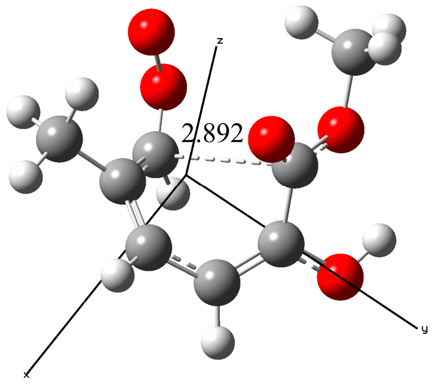

IM9

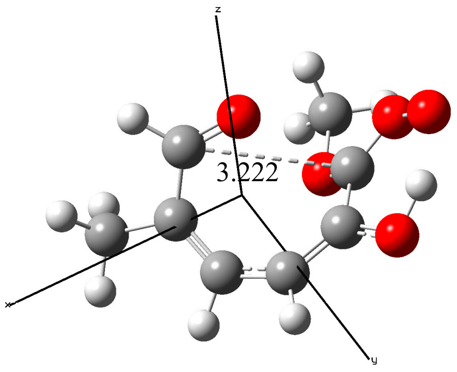

IM10

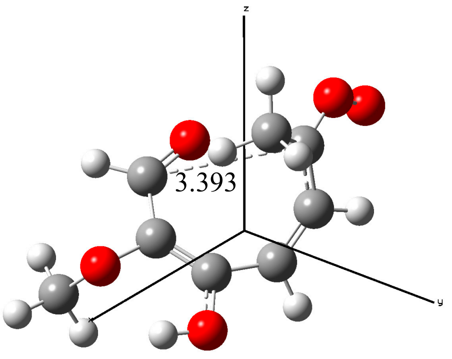

IM11

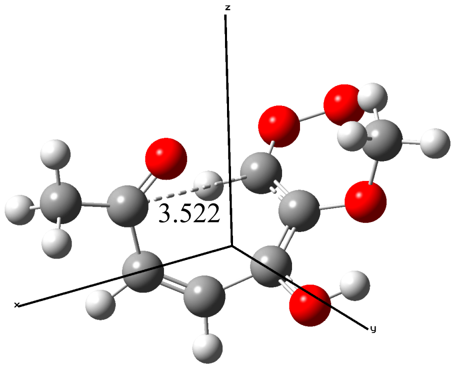

IM12

Figure S10. Continued.

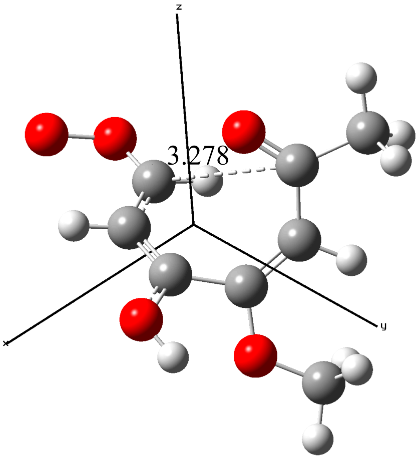

IM13

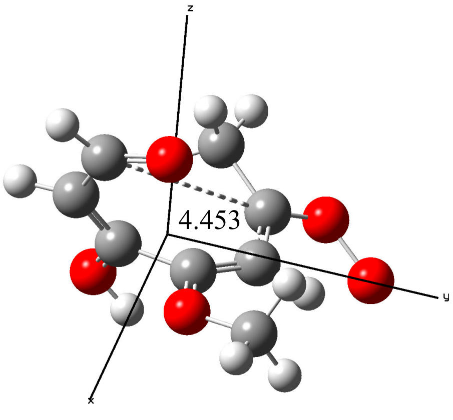

IM14

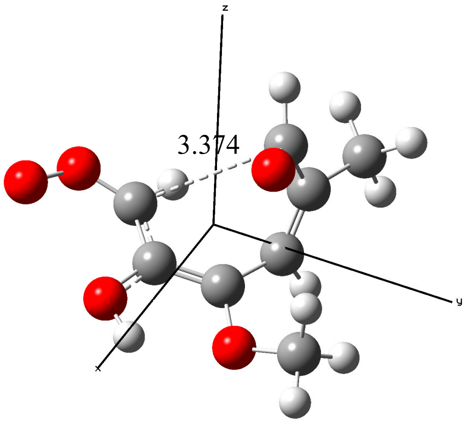

IM15

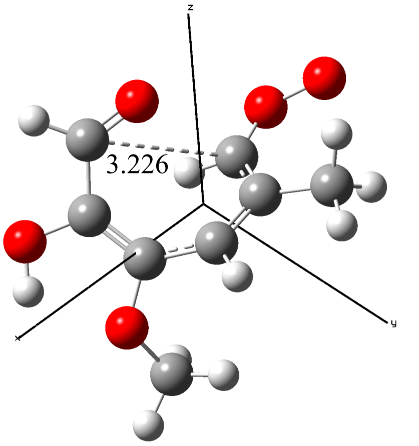

IM16

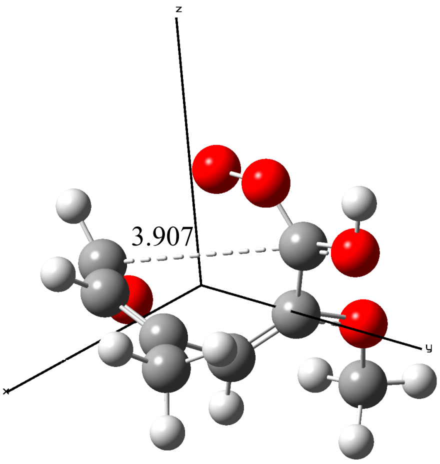

IM17

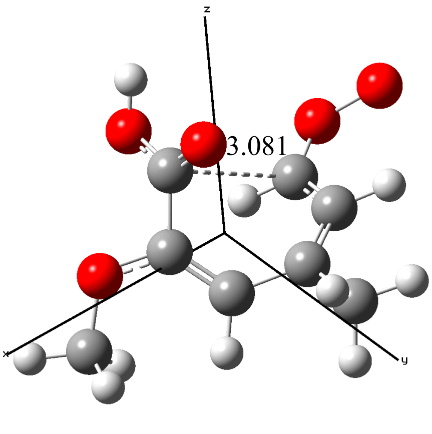

IM18

Figure S10. Continued.

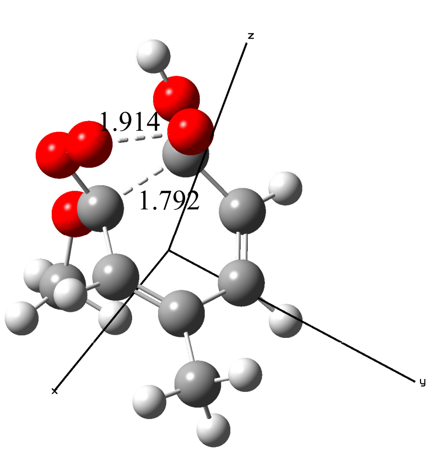

TS7

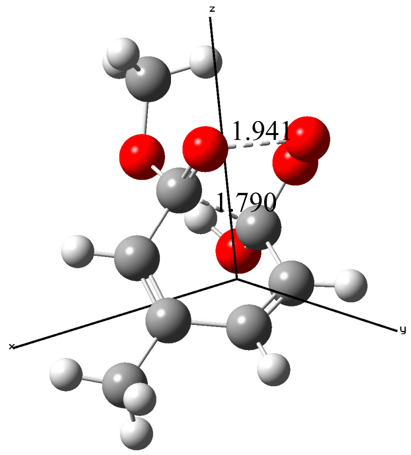

TS8

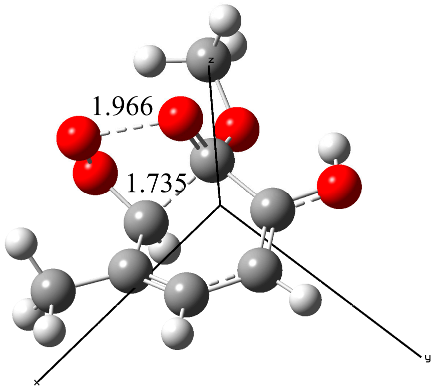

TS9

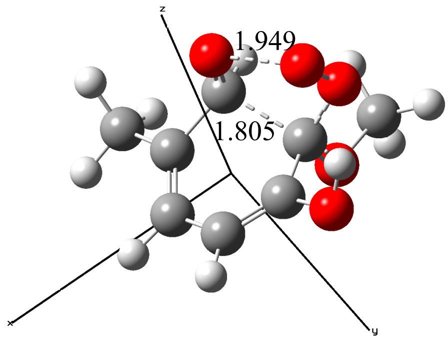

TS10

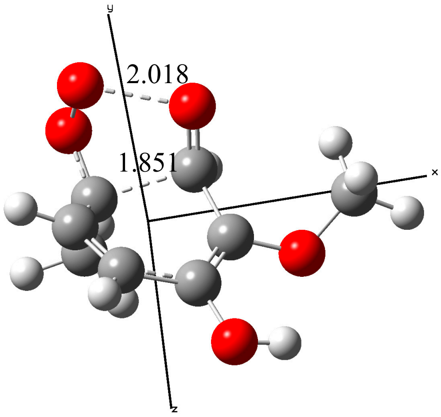

TS11

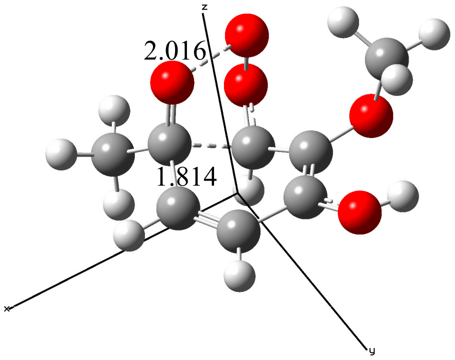

TS12

Figure S10. Continued.

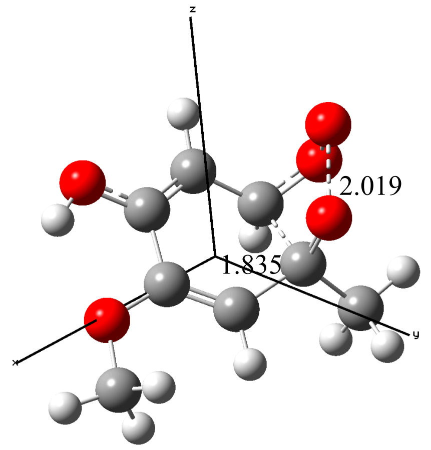

TS13

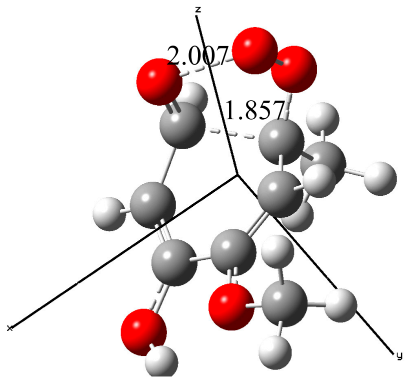

TS14

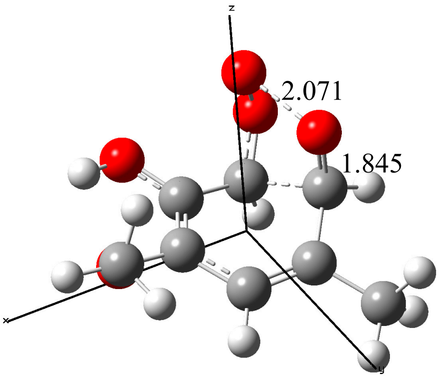

TS15

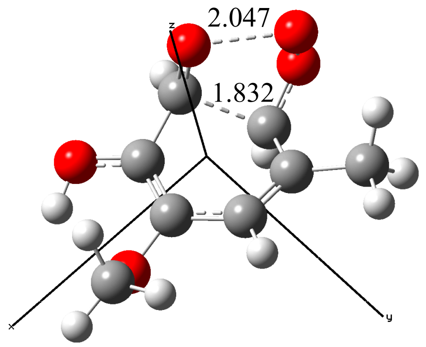

TS16

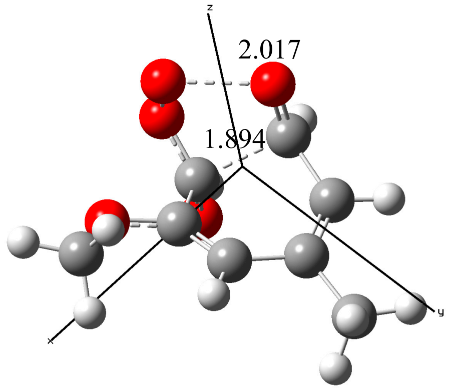

TS17

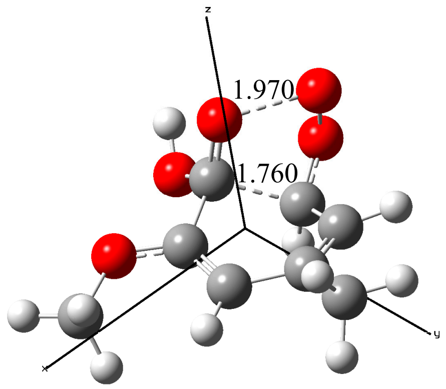

TS18

Figure S10. Continued.
